# Indoloquinoline Ligands Favor Intercalation at Quadruplex‐Duplex Interfaces

**DOI:** 10.1002/chem.202103718

**Published:** 2022-01-05

**Authors:** Yoanes Maria Vianney, Klaus Weisz

**Affiliations:** ^1^ Institute of Biochemistry Universität Greifswald Felix-Hausdorff-Str. 4 17489 Greifswald Germany

**Keywords:** indoloquinolines, intercalation, isothermal titration calorimetry, NMR spectroscopy, quadruplex-duplex junctions

## Abstract

Quadruplex‐duplex (Q‐D) junctions are increasingly considered promising targets for medicinal and technological applications. Here, a Q‐D hybrid with a hairpin‐type snapback loop coaxially stacked onto the quadruplex 3’‐outer tetrad was designed and employed as a target structure for the indoloquinoline ligand SYUIQ‐5. NMR spectral analysis demonstrated high‐affinity binding of the ligand at the quadruplex‐duplex interface with association constants determined by isothermal titration calorimetry of about 10^7^ M^−1^ and large exothermicities Δ*H*° of −14 kcal/mol in a 120 mM K^+^ buffer at 40 °C. Determination of the ligand‐bound hybrid structure revealed intercalation of SYUIQ‐5 between 3’‐outer tetrad and the neighboring CG base pair, maximizing π–π stacking as well as electrostatic interactions with guanine carbonyl groups in close vicinity to the positively charged protonated quinoline nitrogen of the tetracyclic indoloquinoline. Exhibiting considerable flexibility, the SYUIQ‐5 sidechain resides in the duplex minor groove. Based on comparative binding studies with the non‐substituted *N*5‐methylated indoloquinoline cryptolepine, the sidechain is suggested to confer additional affinity and to fix the alignment of the intercalated indoloquinoline aromatic core. However, selectivity for the Q‐D junction mostly relies on the geometry and charge distribution of the indoloquinoline ring system. The presented results are expected to provide valuable guidelines for the design of ligands specifically targeting Q‐D interfaces.

## Graphical abstract

**Quadruplex‐duplex junctions**: A quadruplex‐duplex interface outcompetes an exposed 5’‐outer tetrad of a parallel quadruplex as favored binding site for indoloquinoline ligands. The ligands intercalate between the G‐tetrad and the neighboring Watson‐Crick base pair located at the interface. High‐affinity binding associated with a large exothermicity is driven by maximizing π–π stacking and electrostatic interactions of the positively charged indoloquinoline moiety.

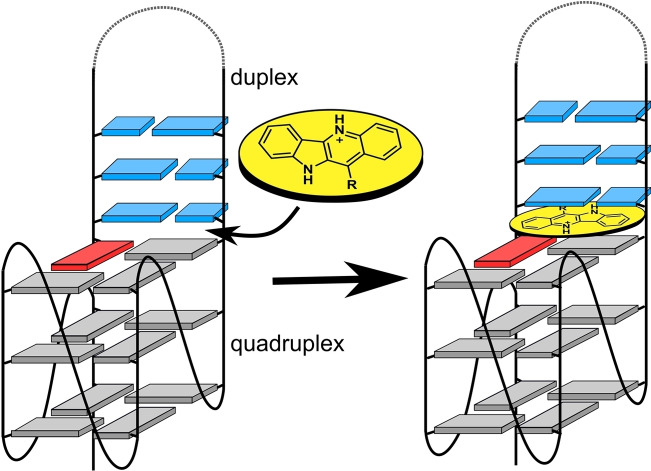

## Introduction

G‐rich sequences are able to fold into four‐stranded quadruplex structures, exerting important biological roles in the regulation of various physiological processes but also constituting powerful tools for an increasing number of technological applications. It has been pointed out that quadruplex formation in the genome may entail the presence of Q‐D junctions through the Watson‐Crick pairing within an appropriate loop element or between a flanking sequence with the single‐stranded complementary strand.[^1^, ^2^] In fact, several natural and designed quadruplex‐forming sequences fold to feature Q‐D interfaces by having loops, bulges, or flanking sequences able to self‐associate into a duplex hairpin.[^3^, ^4^, ^5^, ^6^] Upon the engineering of quadruplex scaffolds, duplex extensions in quadruplexes were shown to promote quadruplex folding or to drive folding into defined quadruplex topologies.[^7^, ^8^, ^9^, ^10^] Also, RNA Q‐D junctions were reported to be specifically recognized by the human fragile X mental retardation RGG peptide[^11^, ^12^] and anti‐thrombotic quadruplexes featuring Q‐D interfaces have demonstrated their great potency as biomedical aptamers.[^13^, ^14^] Consequently, Q‐D junctions have started to become attractive candidates as therapeutic targets but also as novel structural motifs with promising properties. Initial strategies for the design of ligands with a binding propensity for Q‐D hybrid structures are based on linking quadruplex‐binding ligands composed of extended aromatic ring systems for efficient tetrad stacking with typical duplex minor groove binders for the dual binding of both quadruplex and duplex domains.[^2^, ^15^] However, Q‐D junctions themselves have not been exploited for a systematic drug targeting to date, suffering from a paucity of detailed structural information.

**Figure 1:**
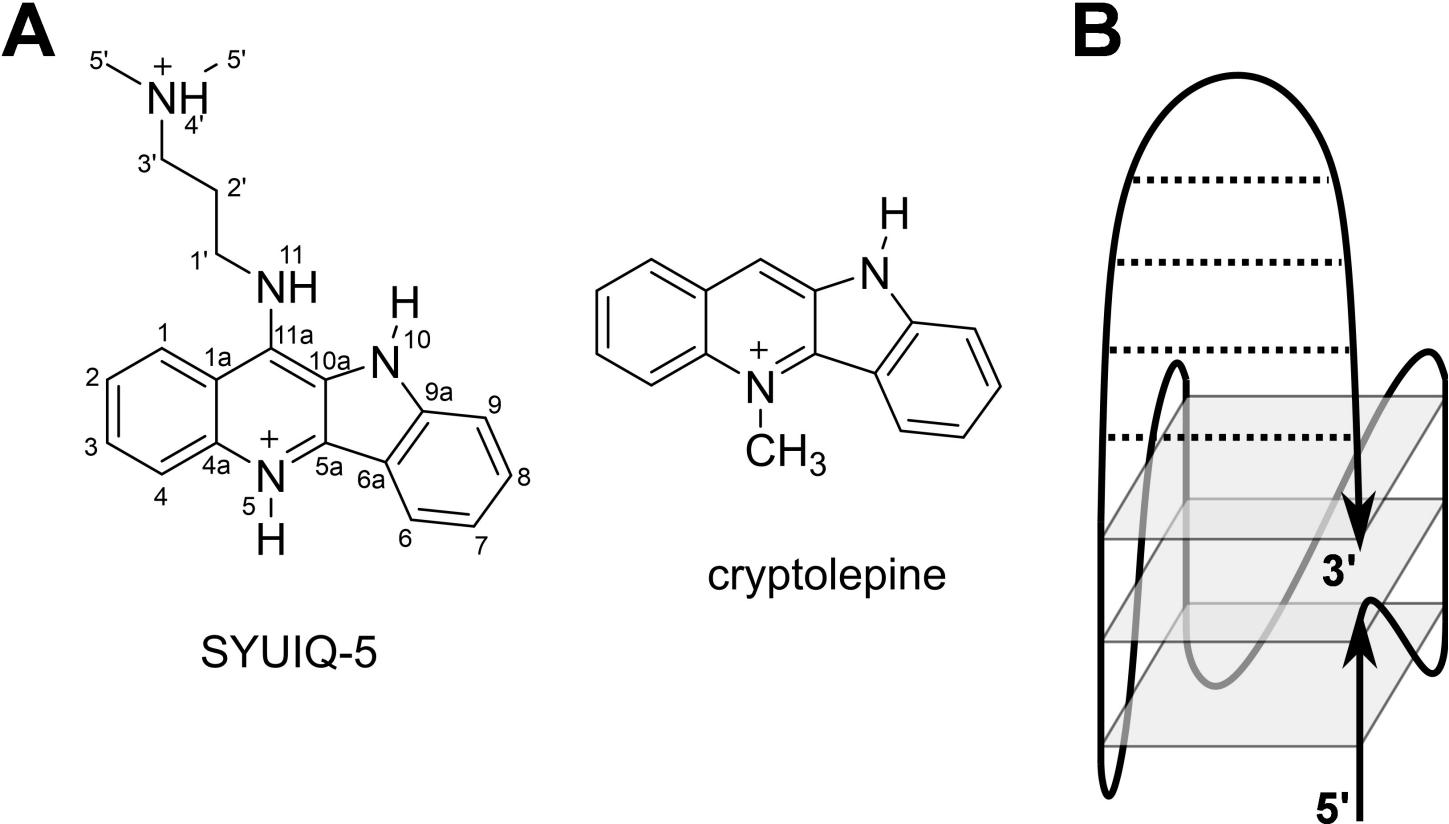
(A) Chemical structure of indoloquinoline derivatives cryptolepine and SYUIQ‐5 with atom numbering. (B) Designed quadruplex topology with a Q‐D junction.

Natural and artificial compounds that are based on the tetracyclic indoloquinoline scaffold possess a wide range of different biological activities (Figure 1A).^[16]^ Thus, the natural alkaloid cryptolepine has long been used as an antimalarial agent.^[17]^ In addition, various indoloquinoline derivatives are known to be potent binders to DNA structures and in particular to G‐quadruplexes.[^18^, ^19^] These include the anticancer drug SYUIQ‐5, shown to exhibit telomerase inhibition activity.[^20^, ^21^] The binding of a closely related indoloquinoline to the parallel *c‐Myc* quadruplex has been structurally and thermodynamically characterized in detail.[^22^, ^23^] As for other typical quadruplex ligands, the planar indoloquinoline ring system binds through end‐stacking onto the exposed 5’‐ and 3’‐outer tetrad, additionally fixed through the formation of a binding pocket involving short overhang sequences. Binding is mostly driven by favorable stacking interactions and hydrophobic effects. In trying to optimize ligands for improved biological activities and better quadruplex affinities and selectivities, substitution patterns and ligand sidechains have been modified to provide a large number of indoloquinoline derivatives during the past two decades.[^24^, ^25^, ^26^] In fact, indoloquinoline ligands with appropriate sidechains have been shown to not only increase affinities due to additional complex‐stabilizing interactions but to also favor quadruplex over duplex binding and to discriminate among different quadruplex folds.[^25^, ^26^] However, whereas the impact of sidechains on the binding thermodynamics can easily be evaluated, local sidechain interactions have been difficult to pinpoint in most cases owing to their considerable flexibility.

Building upon the adaptable and promising DNA binding properties of the indoloquinoline scaffold, we recently reported on the binding of an 11‐phenyl substituted indoloquinoline derivative to a hybrid structure with a Q‐D junction formed by a dangling 3’‐hairpin extending from a parallel quadruplex.^[27]^ A favored enthalpy‐driven binding at the Q‐D junction could be demonstrated, however, NMR experimental limitations precluded the determination of a high‐resolution structure with a well‐defined ligand binding site. Therefore, a modified Q‐D hybrid was designed to be used as a target for the indoloquinoline SYUIQ‐5 in the present study (Figure 1B). The hybrid was constructed from a typical parallel G‐quadruplex by extending its 3’‐terminus by a self‐complementary hairpin‐forming sequence. Inspired by various Q‐D hybrids originally engineered by Phan,^[28]^ the duplex stem‐loop was additionally fixed to the G‐core through a 3’‐terminal G, filling a vacant site of the quadruplex outer tetrad. Such a model architecture is expected to decrease flexibilities and to yield a better defined Q‐D junction for structure determinations, yet may nevertheless mimic parallel quadruplexes with a coaxially stacked duplex as a potential target in promoter regions of oncogenes. As demonstrated by NMR experiments, SYUIQ‐5 with its aminoalkyl sidechain binds the Q‐D junction with high affinity. The three‐dimensional solution structure of the major 1 : 1 complex reveals ligand intercalation between the outer G‐tetrad and the adjacent duplex base pair. Additional binding studies with unsubstituted cryptolepine give further insight into the impact of the sidechain on the indoloquinoline binding.

## Results

### Structure and stability of the *QD3‐sbl* hybrid

The 36mer oligonucleotide *QD3‐sbl* is based on the parallel‐folded *c‐Myc* quadruplex^[29]^ with a 3’‐flanking Watson‐Crick self‐complementary sequence and a 5’‐TTA overhang found to exhibit cleaner spectra (Table S1). Also, featuring only a truncated first GG run, a 3’‐terminal guanine base appended to the hairpin domain was expected to dock into the last vacant position of the first G column of the parallel fold to result in a duplex stem‐loop fixed at both of its ends to the 3’‐outer tetrad of the quadruplex (Figure 1B). In the following, NMR and thermal melting experiments were performed in a buffer solution with 10 mM potassium phosphate, pH 7. With no noticeable structural change when compared to an environment with higher K^+^ concentrations (not shown), these low‐salt conditions allowed for the observation of melting temperatures within a convenient temperature range and also yielded an improved sensitivity in NMR experiments.

**Figure 2:**
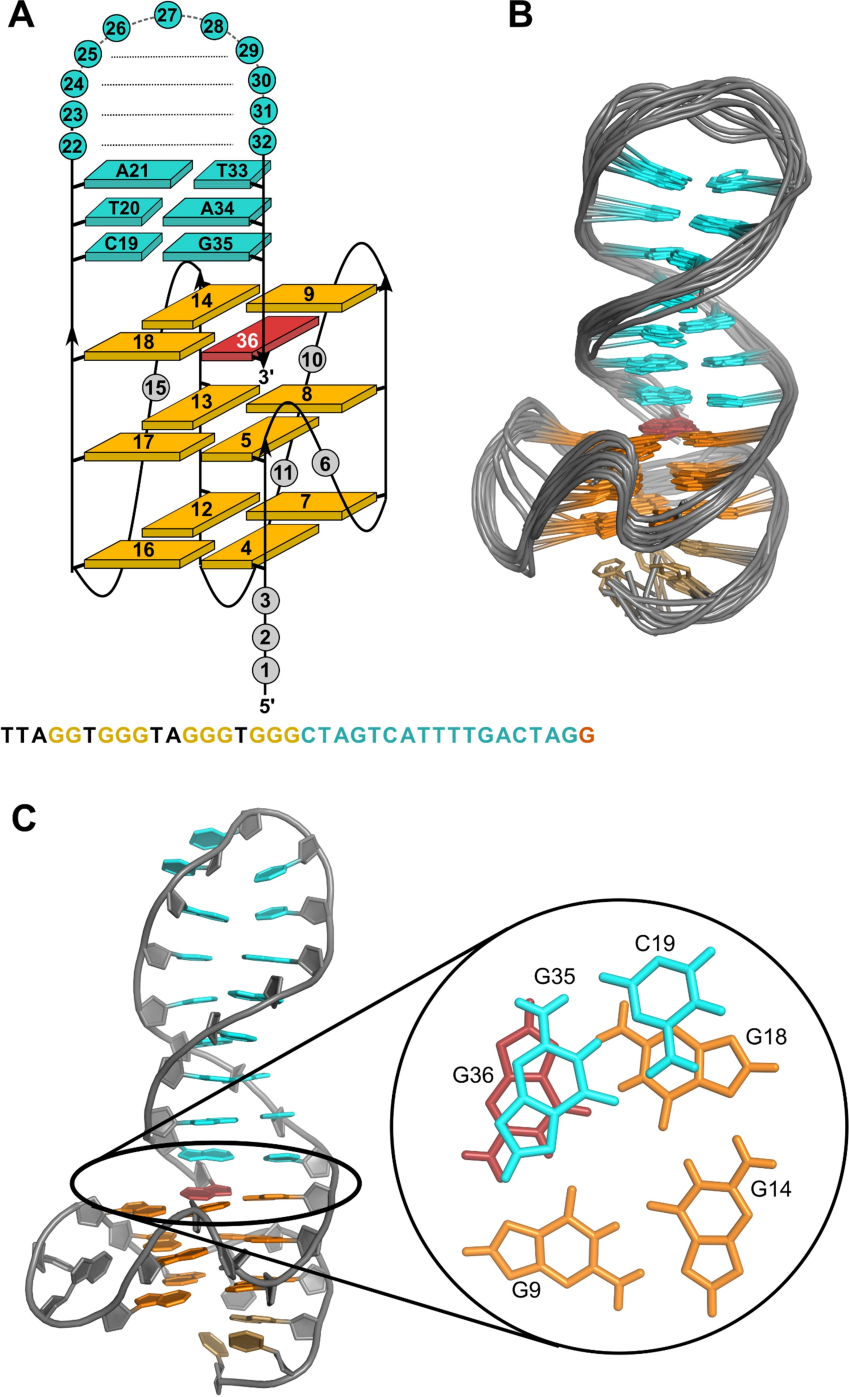
(A) Schematic representation of the *QD3‐sbl* hybrid structure with oligonucleotide sequence. (B) Superposition of ten lowest‐energy structures of *QD3‐sbl*; residues in the quadruplex propeller and the hairpin T_3_‐loops are omitted for clarity. (C) Representative structure of *QD3‐sbl* with a close‐up view of the C19⋅G35 base pair stacked onto the outer tetrad at the Q‐D junction; *anti*‐G residues of the tetrad, *syn*‐G36, and residues of the stem‐loop are colored orange, red, and cyan, respectively.

The imino proton NMR spectral region of a *QD3‐sbl* buffer solution suggests a well‐defined structure with Hoogsteen G imino resonances between 10.6 and 12.0 ppm indicative of a three‐layered quadruplex and additional more downfield shifted Watson‐Crick imino protons through duplex formation between 12.6 and 13.8 ppm (Figure S1A). Resonance assignments of the *QD3‐sbl* hybrid were facilitated by strong correspondences of NOE contacts in expected quadruplex and duplex domains with NOE patterns previously found for a closely related quadruplex‐duplex hybrid but with non‐interrupted G‐tracts and a loose duplex 3’‐terminus.^[27]^ Sequential H8/H6‐sugar NOE walks from 5‘‐terminal T1 to the 3‘‐penultimate G35 also include T26–T28 of the putative hairpin loop and are only interrupted at propeller loop residues T6, T10, and T15 (Figure 2A, S1B). Noticeably, 3‘‐terminal G36 lacks sequential contacts to G35 but has cross‐peaks to G5 of the first GG‐tract as expected when filling the vacant position in the 3‘‐outer G‐tetrad. Also, its *syn* glycosidic torsion angle is shown by a strong intra‐nucleotide H8−H1’ cross‐peak and a rather downfield‐shifted ^13^C8 in HSQC spectra (Figure S1B, S2). Homo‐polarity of all tetrads with hydrogen bonds running into the same direction is demonstrated by typical H8−H1 connectivities within the quadruplex core composed of an *anti*‐*anti*‐*syn* column for G4−G5−G36 and three all‐*anti* columns for G7−G8−G9, G12−G13−G14, and G16−G17−G18 (Figure 2A, S1C). Characteristic imino‐imino contacts within the G‐core further corroborate the alignment of G residues in the parallel quadruplex (Figure S1A).

Imino protons of the duplex base pairs were assigned according to standard strategies making use of their NOE contacts to cytosine amino and adenine H2 protons. All seven Watson‐Crick base‐paired imino protons of thymine and guanine bases could be identified. Notably, in addition to the A25⋅T29 base pair following the flexible T_3_ hairpin loop, imino protons of T20 and in particular G35 in the two base pairs bordering the quadruplex‐duplex junction are broadened and of low intensity. This suggests enhanced dynamics at the junction with its anchored 3’‐terminal *syn*‐G36. However, a strong conspicuous contact from G35 H8 to G36 H1 demonstrates continuous stacking of the duplex stem‐loop onto the 3’‐tetrad (Figure S1C). On the opposite face of the G‐core, various cross‐peaks connect the short 5’‐overhang with the neighboring 5’‐outer tetrad. Based on cross‐peak patterns in DQF‐COSY spectra with large H1’−H2’ scalar couplings, 26 residues were unambiguously found to adopt sugar puckers in the pseudorotational south domain (Figure S3).

Structure calculations employed NMR‐derived distance and dihedral angle restraints (statistics and a list of chemical shifts are given in Table S2 and S3). Final structures feature a quadruplex‐duplex hybrid composed of a parallel three‐layered quadruplex with a broken first G‐column (Figure 2). A double‐helical stem‐loop with its seven Watson‐Crick base pairs extends from the fourth G‐tract with coaxial stacking of the duplex onto the quadruplex domain and is additionally fixed by the appended 3’‐terminal *syn*‐guanosine that fills an empty G‐core position. In fact, the hairpin‐type 3’‐overhang can be regarded a double‐helical lateral snapback loop. Whereas residues of the G‐core and the Watson‐Crick base pairs are well defined, residues in the 5’‐overhang, in the quadruplex propeller loops, and in the hairpin T_3_‐loop are more flexible (Figure 2B). With the duplex connecting adjacent edges of the outer tetrad, its minor groove follows the quadruplex groove between the first and fourth G‐column while the duplex major groove at the junction faces the center of the G‐tetrad. Efficient stacking interactions are observed between G35 and G36 but C19 stacking onto G18 is only poor (Figure 2C). On the other quadruplex face, A3 of the 5’‐overhang is found to cap G4 and G16 of the 5’‐tetrad in line with corresponding NOESY cross‐peaks (Figure S1B,C).

DSC thermograms revealed two distinct melting transitions for the quadruplex and duplex domains at 40.2 °C and at 47.2 °C in a 10 mM K^+^ buffer solution (Figure S4). Independent melting of the secondary structures was additionally confirmed by UV melting experiments. By analyzing temperature dependent absorbance changes at 260 nm for the duplex and at 295 nm for the quadruplex, melting of the latter could be assigned to the lower melting transition (Table S4). Apparently, despite their coaxial stacking there is no cooperative melting of quadruplex and duplex domains in line with corresponding observations on a quadruplex carrying a 3’‐flanking duplex domain with a dangling terminus.^[27]^

### The Q‐D junction constitutes the preferred ligand binding site

Initial CD titrations indicated that addition of SYUIQ‐5 to *QD3‐sbl* has no significant impact on the Q‐D hybrid structure (Figure S5). On the other hand, binding of the ligand is demonstrated by an induced CD effect (ICD) at the ligand absorption centered at 350 nm. Of note, a small‐amplitude negative ICD compatible with end‐stacking changes to a positive ICD of higher amplitude with ligand in excess. Apparently, at stoichiometries >1 additional ligand interacts with the Q‐D hybrid albeit with weaker affinity, overwriting the initial ICD signature.

**Figure 3:**
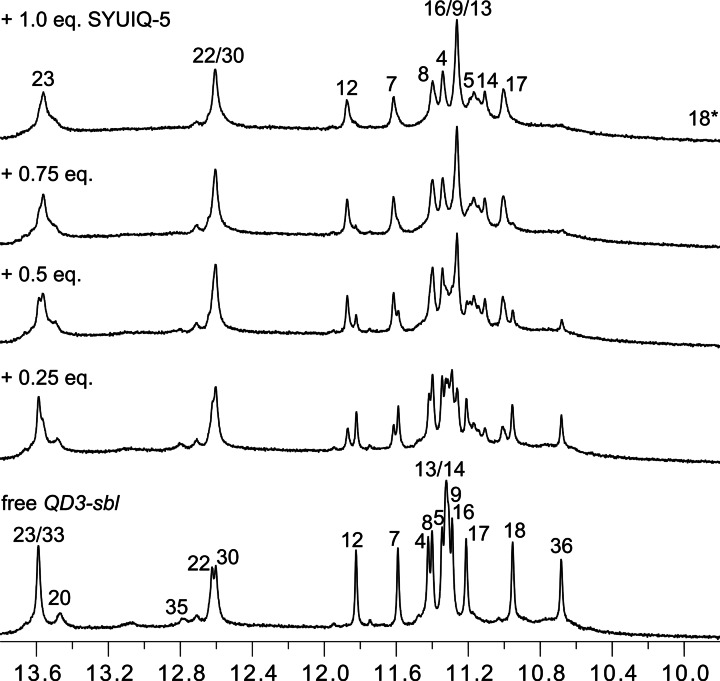
Imino proton spectral region of *QD3‐sbl* (1 mM) titrated with SYUIQ‐5 at 30 °C. Assigned peaks of the free hybrid and the 1 : 1 complex are labeled with residue numbers; note that the marked G18 imino signal of the complex is unobservable in the 1D spectrum but unambiguously assigned through exchange cross‐peaks in NOESY and ROESY spectra.

To yield sharper resonances, subsequent NMR titrations of the ligand to the hybrid were performed at 30 °C. Looking at the imino proton spectral region, additional Hoogsteen G imino signals gradually emerged upon ligand addition with resonances of the free hybrid structure completely lost at a 1 : 1 molar ratio (Figure 3). Coexisting resonances of free and complexed species observed with 0.5 equivalent of ligand indicate their slow exchange. On the other hand, downfield‐shifted Watson‐Crick imino resonances seem to only show modest heterogenous and/or homogenous line broadening effects, suggesting smaller perturbations in the duplex stem‐loop upon initial ligand binding.

**Figure 4:**
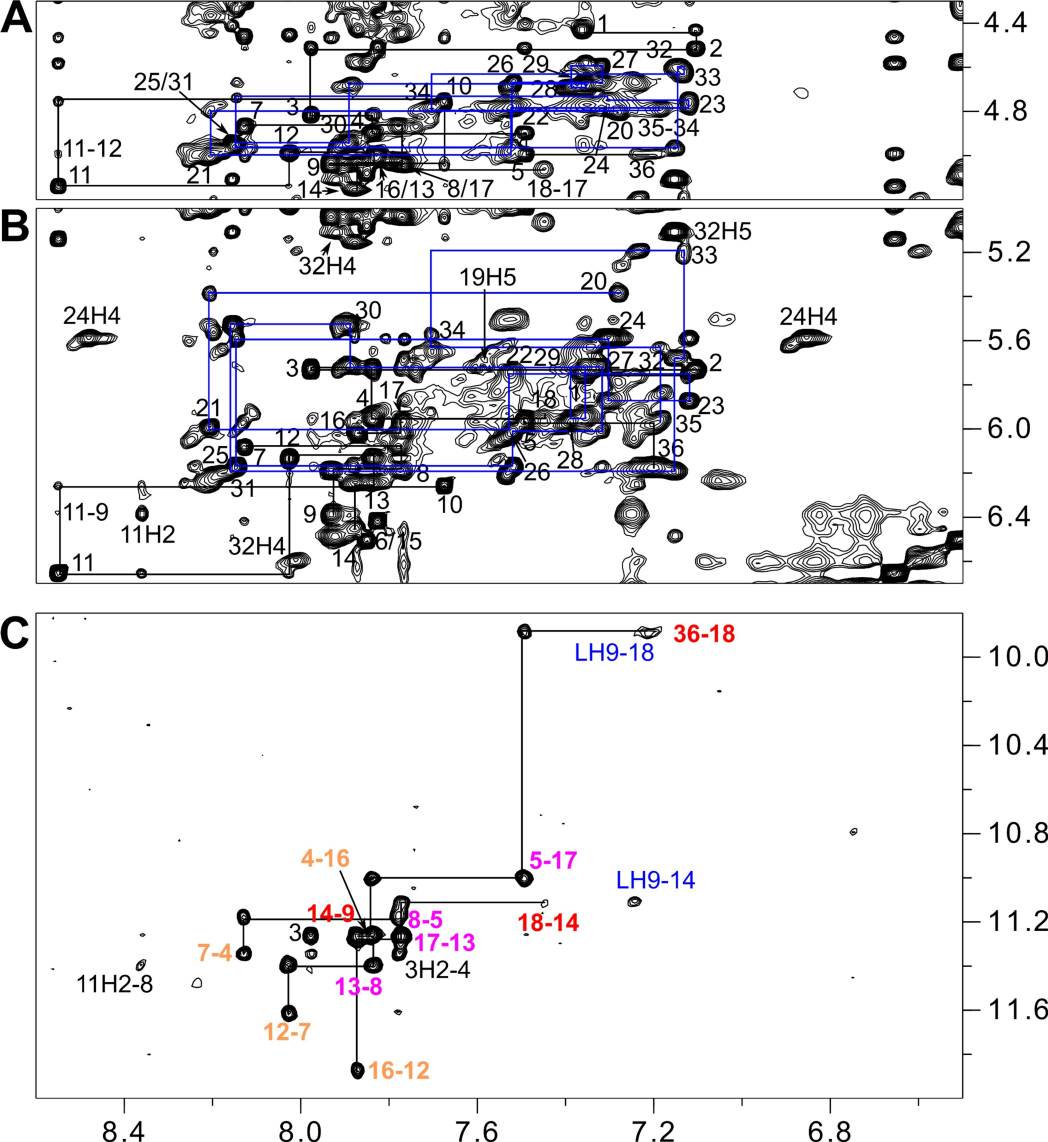
Regions of a 2D NOESY spectrum (300 ms mixing time, 30 °C) of *QD3‐sbl* (1 mM) in the presence of 1 equiv. SYUIQ‐5. (A) H6/8(ω_2_)−H3’(ω_1_) and (B) H6/8(ω_2_)−H1’(ω_1_) spectral region; continuous networks of base‐sugar resonances are followed by vertical and horizontal lines with NOE connectivities in the duplex domain traced by blue lines; intra‐nucleotide cross‐peaks are labeled by residue number. (C) H8/6/2(ω_2_)−H1(ω_1_) spectral region; intra‐tetrad H8(ω_2_)−H1(ω_1_) cross‐peaks are labeled with colors depending on G‐tetrad layer; inter‐tetrad connectivities along the G‐columns are traced by horizontal and vertical lines and intermolecular contacts between quadruplex imino and ligand protons are labeled in blue.

Supported by a close analogy to the already assigned free Q‐D hybrid, standard strategies involving NOESY, DQF‐COSY, and ^1^H‐^13^C HSQC experiments were again used to identify non‐labile protons in the 1 : 1 complex. Thus, continuous sequential NOE connectivities between H8/H6 and sugar protons, interrupted by the propeller loops, allowed for the assignment of most non‐exchangeable proton resonances (Figure 4A,B, S6A). Again, 3’‐terminal G36 features characteristic NOE contacts to G5 of the first G‐tract. However, lost connectivities between G18 and C19 at the Q‐D interface adds another interruption to the sequential NOE walk in the ligand‐bound hybrid.

Noticeable homogenous and/or heterogenous broadening of resonances at the Q‐D junction hampered the unambiguous resonance assignment for affected residues, yet support from sequential H8/6−H8/6 contacts, ^1^H‐^13^C HSQC, and DQF‐COSY spectra enabled spectral identification for most protons (Figure S6A, S7, S8). It should be mentioned that some broadening of H8/6−H1’ cross‐peaks from duplex nucleotides not only depends on their vicinity to the junction but shows an asymmetric behavior along the duplex stem‐loop. In contrast to the 5’‐terminal hairpin strand extending from the quadruplex core, line broadening effects further continue along the complementary duplex strand beyond interfacial G35 to A34 and up to T33. Finally, if accessible through resolved H1’(ω_2_)−H2’/H2”(ω_1_) DQF‐COSY cross‐peak patterns and NOESY contacts at short mixing times, the sugar pucker of residues could unambiguously be assigned to a south conformation (Figure S8).

Except for the G36 imino, assignment of all other imino resonances of the G‐core was enabled through H8−H1 NOE contacts and additionally supported by characteristic imino‐imino connectivities as well as by ROESY exchange cross‐peaks observed between the free and complexed Q‐D hybrid after the addition of 0.5 equivalent of ligand (Figure 4C, S6B, S9). Imino connectivities also demonstrated formation of a three‐layered parallel quadruplex core with a counter‐clockwise direction of Hoogsteen hydrogen bonds within G‐quartets in line with no major structural rearrangements upon ligand binding. A conspicuous upfield shift of more than 1 ppm for the G18 imino proton after complex formation, confirmed by a corresponding exchange cross‐peak in a ROESY experiment (Figure S9), again hints at a ligand binding site at the Q‐D junction. Due to a significant ligand‐induced broadening of duplex imino resonances especially for residues near the junction and for the base pair following the hairpin loop, only imino protons of centrally located base pairs could unambiguously be assigned through their strong contacts to cytosine H4 or adenine H2 protons (Figure S6C).

Protons of the ligand were assigned based on a combination of DQF‐COSY, TOCSY, and NOESY experiments (Figure S10). Amino and aliphatic protons of the SYUIQ‐5 sidechain could be traced through their vicinal and long‐range couplings as observed in DQF‐COSY and TOCSY spectra (Figure S10A). Similarly, correlations in DQF‐COSY spectra also enabled assignments of the scalar coupled aromatic protons of quinoline and indole subunits (Figure S10B). A contact from a ligand H2’ aliphatic proton to the quinoline moiety of the indoloquinoline discriminates the quinoline and indole ring system. Fast exchange prevented observation of NH protons for the indole N10 and quinoline N5. Given a p*K*_a_ of 8.4,^[20]^ the latter is expected to be protonated even in more hydrophobic environments. Due to the absence of contacts between these exchangeable NH protons to other protons of the indole ring system, unambiguous assignments to H6/H7 and H9/H8 proton pairs on the two sides of the fused benzene ring were hampered. However, intermolecular NOE connectivities of indole and quinoline with *QD3‐sbl* resonances in the complex enabled a discrimination of these non‐labile indole protons. A compilation of chemical shifts for *QD3‐sbl* and SYUIQ‐5 in the complex are given in Table S5 and S6.

A total of 11 intermolecular NOE contacts to the Q‐D hybrid position the ligand within the complex (Table S7). These include cross‐peaks of ligand quinoline or indole protons to non‐exchangeable protons of G18, C19, and G35 at the Q‐D interface but also to imino protons of residues G9 and G14 at the exposed edge of the 3’‐tetrad (Figure 4C, S11). No contact to G36 could be observed likely due to dynamic processes. Additional non‐observable intermolecular contacts between indole H9 and G14 sugar protons with their rather sharp signals were added as repulsion restraints in subsequent structure calculations. While the absence of any intermolecular NOE contact of the dimethylamino group attests to a high flexibility of the ligand sidechain, a single contact was detected from ligand aliphatic H2’a/b protons (restrained as C2’) to the G5 H2” sugar proton in the quadruplex groove just below the Q‐D junction. Two unexpected NOE contacts incompatible with the other short intermolecular distances connect aromatic protons of the ligand with a sugar proton of residue T33 located three bases off the junction and also to A3 H8 located at the opposite face of the G‐core (not shown). The latter observations suggest that in addition to a major binding site there may be some minor binding of the ligand within the duplex domain and also onto the 5’‐outer tetrad.

### Solution structure of the 1:1 complex

**Figure 5:**
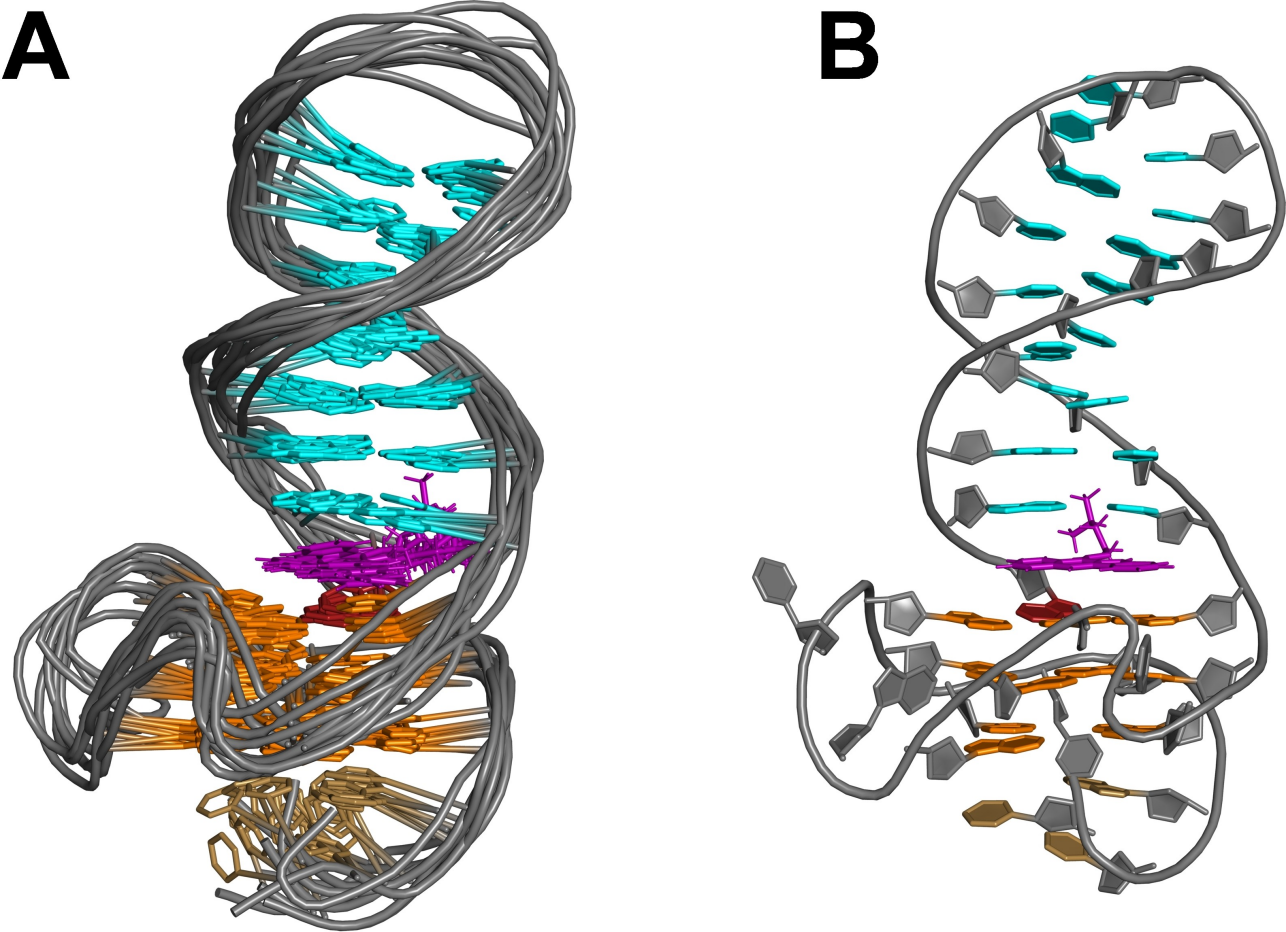
Side view of (A) ten superimposed lowest‐energy structures and (B) a representative structure of a 1 : 1 complex between *QD3‐sbl* and SYUIQ‐5. Bases in the quadruplex propeller and hairpin T_3_ loop are omitted for clarity in (A). *Syn*‐guanosines, *anti*‐guanosines, duplex bases, and ligand are colored red, orange, cyan, and magenta, respectively.

A superposition of ten calculated lowest‐energy structures shows a good convergence of the 1 : 1 complex (Figure 5A, Table S3). Despite the limited number of unambiguously assigned intermolecular contacts used as restraints in the structure calculations, the quadruplex‐duplex junction with the bound indoloquinoline aromatic ring system is well defined. A single distance violation >0.2 Å in 1 out of 10 structures applies to contacts between the 5’‐overhang and the 5’‐tetrad and is thus far removed from the SYUIQ‐5 binding site. The ligand intercalates between G18 and G36 of the 3’‐outer tetrad and the C19 ⋅ G35 Watson‐Crick base pair at the Q‐D junction. As a consequence of the ligand insertion between G‐tetrad and base pair, the helical rise at the Q‐D junction increases to 7 Å but also results in some conformational adjustments. Compared to the arrangement in free *QD3‐sbl*, the stacked duplex stem‐loop is shifted towards the center of the G‐tetrad with interfacial C19 positioned above G18 (Figure S12). The ligand sidechain faces the minor groove of the duplex as already suggested by the NOESY data. However, poor convergence indicative of high flexibility is observed for the aliphatic substituent and in particular for the protonated dimethylamino group.

**Figure 6:**
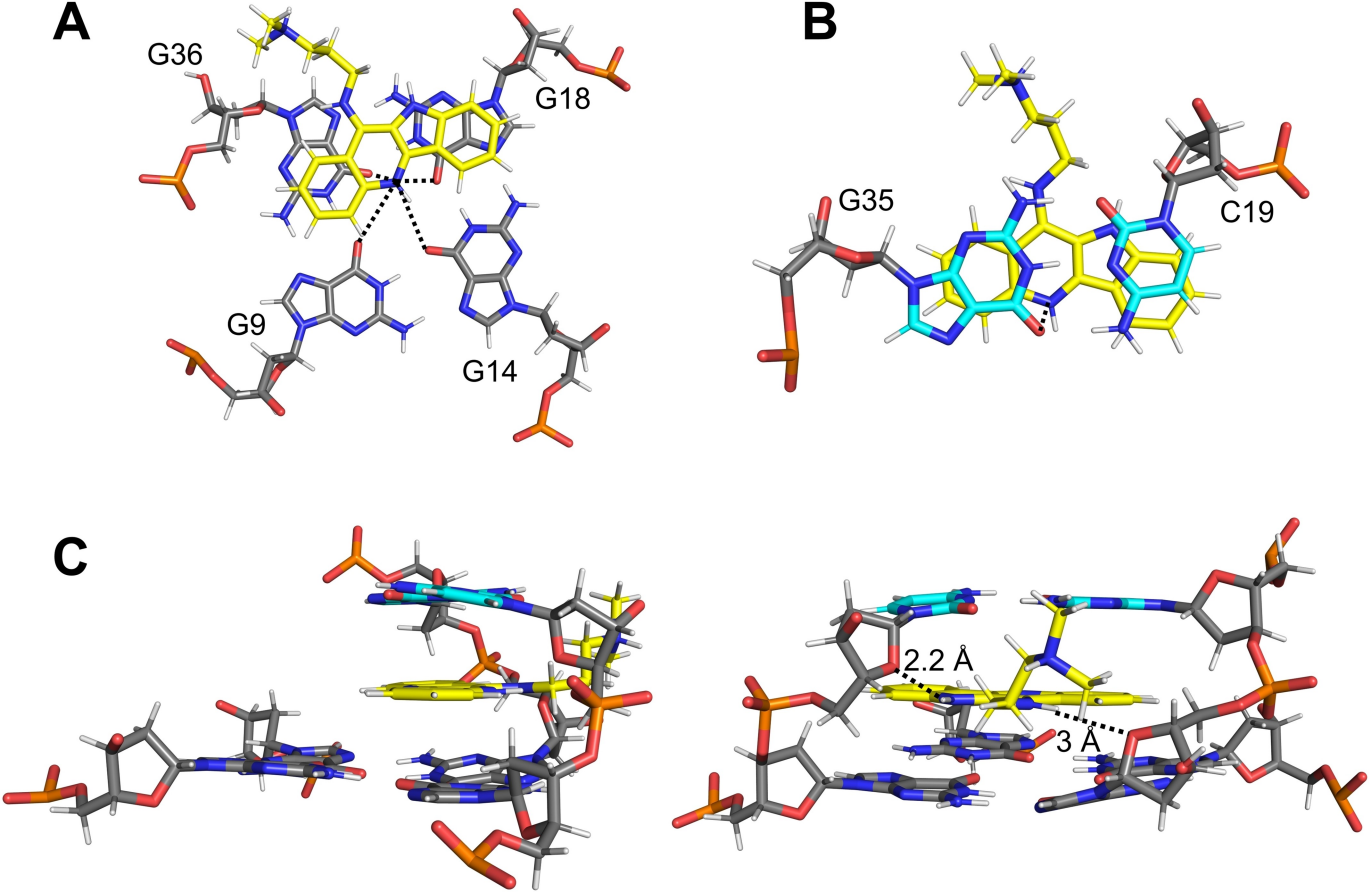
Stacking of the ligand (A) onto the 3’‐tetrad and (B) below the G35⋅C19 Watson‐Crick base pair; distances between the protonated ligand *N*5 and adjacent guanine carbonyl atoms are indicated by dotted lines. (C) Side view (left) and view into the minor groove (right) showing the Q‐D junction with intercalated ligand; short NH10‐C19 O4’ and NH11‐G36 O4’ distances are indicated by dotted lines. The carbon skeleton of the ligand is colored yellow.

The lateral shift of the duplex towards the center of the quadruplex is accompanied by more efficient π–π stacking interactions of the intercalated ligand. Thus, the indoloquinoline tetracyclic ring system is found to insert with the quinoline and indole moieties mostly sandwiched between G36 and G35 and between G18 and C19, respectively. With the protonated and positively charged *N*5 of the indoloquinoline positioned above the central channel of the G‐core at the major groove side of the duplex stem‐loop, additional Coulombic interactions are expected with the four guanine carbonyl oxygen atoms of the 3’‐tetrad but also with G35 of the base pair on top, being in close vicinity with distances ∼4 Å (Figure 6A,B). Potential hydrogen bond interactions with short distances are indicated between indole NH10 and C19 O4’ in 3 out of 10 structures and between NH11 of the sidechain and G36 O4’ in 5 out of 10 structures (Figure 6C). It should be noted that the latter hydrogen bond is only enabled by the antiparallel orientation of docked G36 relative to the other G‐core residues. Given a high flexibility of the ligand sidechain, there is no indication for a hydrogen bond interaction of the terminal dimethylamino group. Yet, electrostatic interactions with the sugar‐phosphate backbone can be assumed.

### Binding of SYUIQ‐5 to an antiparallel quadruplex with a central hairpin lateral loop

**Figure 7:**
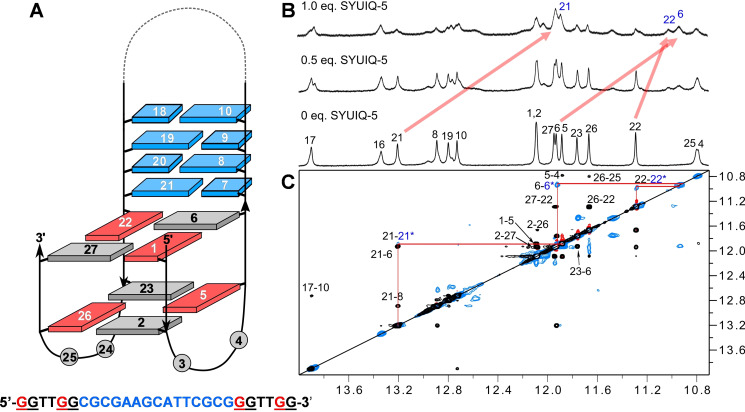
(A) Topology of *QD2‐l* with sequence. (B) Imino proton spectral region upon titrating SYUIQ‐5 to *QD2‐l* (0.5 mM) at 20 °C; most pronounced shifts are indicated by red arrows. (C) Superposition of NOESY spectrum for the free hybrid (black) and ROESY spectrum after the addition of 0.5 equiv. of ligand (blue: positive intensity; red: negative intensity) showing imino(ω_2_)‐imino(ω_1_) correlations; red lines connect most shifted proton resonances at the Q‐D junction as verified through exchange cross‐peaks between the free and complexed *QD2‐l* hybrid. Residue numbers of the free and ligand‐bound hybrid are marked in black and blue, respectively.

To also examine Q‐D junctions as a target in a different structural context, an antiparallel quadruplex termed *QD2‐l*, derived from the thrombin binding aptamer with the second loop modified by a duplex stem‐loop,^[28]^ was employed in additional binding studies with SYUIQ‐5 (Figure 7A). All hydrogen‐bonded imino protons of the duplex, of the two‐layered G‐core, and also of additional T‐T base pairs formed between the first and the third lateral loop were observed in a low‐salt buffer at 20 °C (Figure S13). With only small shifts for some resonances, further analysis of NOESY spectra confirmed a fold as reported previously under our experimental conditions (PDB 2M8Z).

To identify the major SYUIQ‐5 binding site, the ligand was titrated to the *QD2‐l* hybrid while monitoring the imino proton spectral region (Figure 7B). Signal broadening but also the appearance of new signals demonstrated slow exchange between a free and ligand‐bound quadruplex. Exchange cross‐peaks were observed in ROESY spectra on a mixture of the Q‐D hybrid with 0.5 equivalent of ligand and allowed the identification of several imino resonances of the complex (Figure 7C). For Hoogsteen imino signals, most prominent exchange cross‐peaks positioned far off the diagonal and thus correlating resonances with significant chemical shift differences could be traced to G22 and G6 located at the Q‐D interface. The largest chemical shift perturbation was found for the G21 Watson‐Crick imino proton within the interfacial base pair. Apparently, these resonances are subjected to significant upfield shifts upon ligand binding, again in line with strong π–π stacking interactions through SYUIQ‐5 intercalation at the Q‐D junction.

Taken together, Q‐D junctions seem to constitute major high‐affinity binding sites for the SYUIQ‐5 ligand irrespective of the quadruplex topology or external or internal duplex extensions. Intercalation between an outer G‐tetrad and a base pair seems to support a selective high‐affinity binding with ligands featuring a matched shape for optimal stacking interactions.

### Targeting the *QD3‐sbl* hybrid with cryptolepine

In the complex structure with bound SYUIQ‐5, the ligand sidechain mostly resides in the minor groove of the duplex domain. Because of its high flexibility, no major specific interactions of the aminoalkyl group to the Q‐D hybrid could be identified. Nevertheless, van der Waals and electrostatic effects are expected to add to the favorable binding free energy of the indoloquinoline ligand. To examine the impact of the sidechain on the binding selectivity in more detail, the *QD3‐sbl* hybrid was also targeted with the natural indoloquinoline alkaloid cryptolepine (Figure 1A). Lacking any additional sidechain, this *N*5‐methylated indoloquinoline bears a permanent positive charge but is considered a rather poor quadruplex‐binding ligand both because of its modest discrimination against other nucleic acid secondary structures including duplexes and because of only moderate affinities for G‐quadruplexes.^[16]^

**Figure 8:**
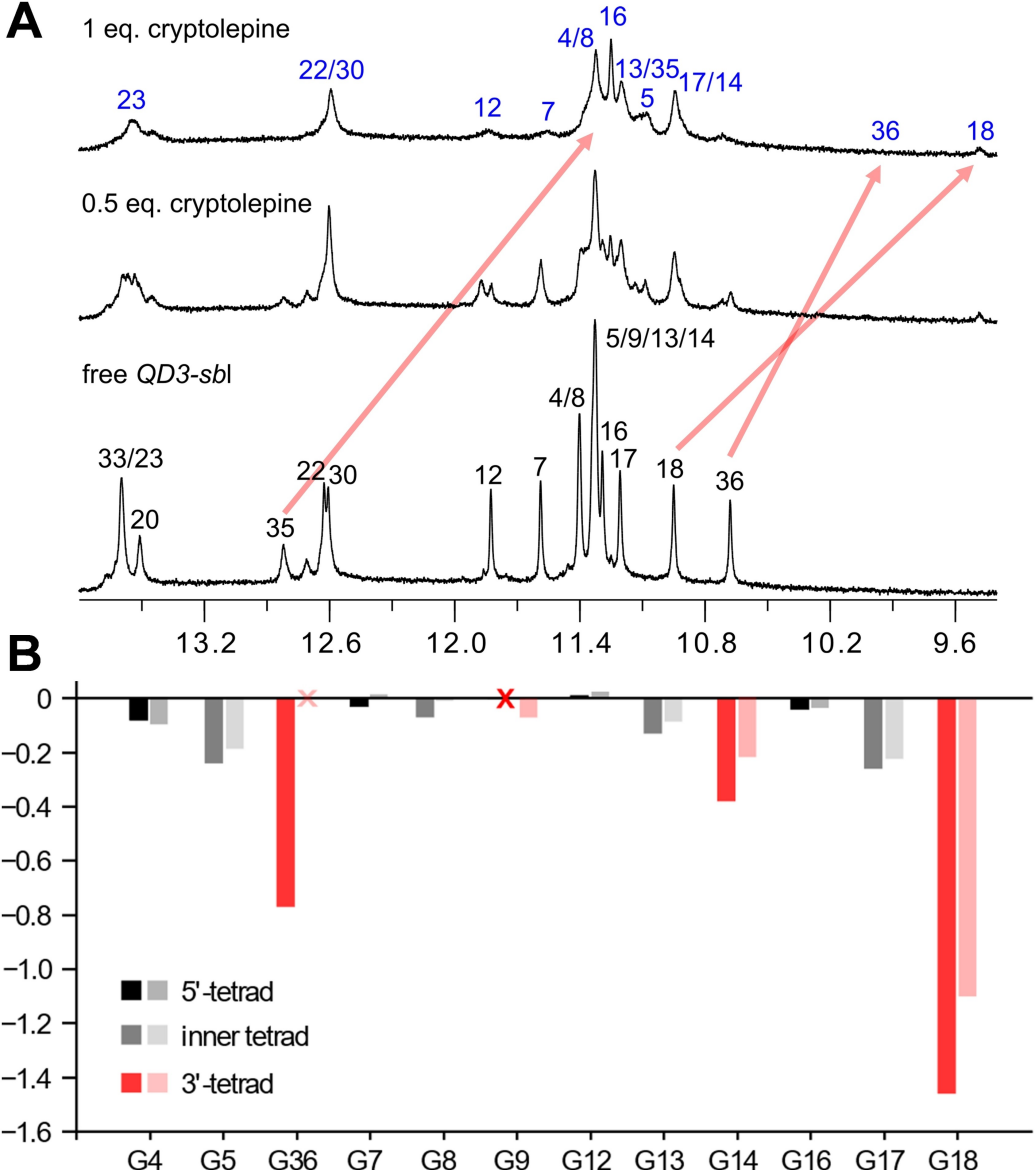
(A) Imino proton spectral region upon titrating cryptolepine to the *QD3‐sbl* hybrid structure (0.5 mM) at 30 °C; most pronounced chemical shift changes for interfacial G‐tetrad and base pair imino protons, corroborated by exchange cross‐peaks in ROESY spectra, are indicated by red arrows. (B) Quadruplex imino proton chemical shift differences between major ligand‐bound and free *QD3‐sbl*; dark‐ and light‐colored bars represent footprints with cryptolepine and SYUIQ‐5, respectively. Red crosses mark non‐assigned imino resonances.

NMR titrations showed signal broadening and the appearance of new resonances upon cryptolepine addition with slowly exchanging free and bound species at ligand‐to‐DNA molar ratios <1 (Figure 8A). Assignments of non‐labile protons in the complex are mostly based on NOESY experiments (Figure S14A). In general, cross‐peak patterns of the NOESY spectrum resemble *QD3‐sbl* when complexed with SYUIQ‐5. However, although continuous base‐sugar NOE connectivities can be traced along the duplex stem‐loop, broadening of cross‐peaks increases towards the Q‐D junction and only allow to unambiguously follow sequential NOE walks from T20 to A34. For the quadruplex domain, H8−H1’ connectivities link all residues along the four G‐columns, again featuring more extensive signal broadening for non‐labile protons at the Q‐D junction when compared to the 5’‐tetrad.

Some quadruplex imino proton resonances in the complex were heavily broadened. Nevertheless, almost complete assignments except for G9 H1 were enabled by the observation of ROESY exchange cross‐peaks on samples with ligand‐to‐DNA molar ratios of 0.5 (Figure S15) and further supported by H8−H1 NOESY cross‐peaks for the 1 : 1 complex (Figure S14B). Notably, there are two pairs of prominent exchange cross‐peaks of similar intensity in the ROESY spectrum that correlate G18 H1 as well as G36 H1 at the interfacial 3’‐tetrad of the free hybrid with two ligand‐bound species (Figure S15B). Being most upfield‐shifted in both complexes, these observations suggest cryptolepine binding at identical sites but with different ligand orientation, for example, as a result of a 180° flip of the indoloquinoline within the binding pocket. Exchange processes between differently aligned ligand is expected to further broaden resonances at the cryptolepine binding site. Overall, G imino protons in the 3’‐tetrad at the Q‐D junction experienced significant chemical shift perturbations when compared to those in the inner and 5’‐tetrad. Also, profiles of G imino chemical shift changes on cryptolepine addition closely follow chemical shift footprints on SYUIQ‐5 binding, indicating favored cryptolepine binding again through intercalation at the Q‐D interface as demonstrated for SYUIQ‐5 (Figure 8B). Consequently, it is the indoloquinoline ring system with its particular geometry and electron distribution that seems to favor binding at Q‐D junctions.

### Thermodynamic profiles for indoloquinoline binding at Q‐D interfaces

Isothermal titration calorimetry was employed to evaluate association constants and thermodynamic profiles of SYUIQ‐5 and cryptolepine binding to the *QD3‐sbl* hybrid structure (Figure S16, Table 1). Being closer to physiological conditions, studies on the binding thermodynamics were performed at 40 °C in a 120 mM potassium phosphate buffer. As has already been suggested by the CD titrations of *QD3‐sbl* with SYUIQ‐5 (see above), thermograms of both indoloquinolines exhibit a high‐affinity binding site followed by additional binding events of lower affinity with only a gradual return to baseline for ligand in excess. Notably, high‐ and low‐affinity binding processes are better resolved for the SYUIQ‐5 ligand, indicating its superior binding selectivity. Only focusing on the high‐affinity binding, the association constant as determined by a curve fit based on a model with two independent binding sites amounts to *K*_a_ ∼ 1⋅10^7^ M^−1^ for SYUIQ‐5, more than a factor of three higher when compared to cryptolepine. Also, a strongly exothermic binding for SYUIQ‐5 is counteracted by an unfavorable entropic contribution to binding. In contrast, binding of cryptolepine is driven by a considerably smaller enthalpic contribution with no additional entropic penalty. Clearly, such thermodynamic profiles corroborate the presence of significant sidechain interactions at the expense of a reduced SYUIQ‐5 conformational freedom.

**Table 1:** Binding thermodynamics of SYUIQ‐5 and cryptolepine to target quadruplexes.^[a]^

	N	*K*_a_ [M^−1^]	Δ*H*° [kcal/mol]	Δ*G*°_313_ [kcal/mol]^[b]^	‐*T*Δ*S*° [kcal/mol]^[b]^
SYUIQ‐5 to					
*QD3‐sbl*	0.9±0.1	(1.1±0.3) ⋅ 10^7^	−14.1±0.2	−10.1±0.2	4.0±0.3
*Q3‐sbl* ^[c]^	1.0±0.1	(2.1±0.6) ⋅ 10^6^	−12.2±0.5	−9.0±0.2	3.2±0.4
cryptolepine to					
*QD3‐sbl*	1.0±0.1	(3.3±0.7) ⋅ 10^6^	−8.0±0.1	−9.3±0.1	−1.3±0.2

[a] Average values and standard deviations for the high‐affinity binding site obtained from three independent measurements in 120 mM K^+^ buffer, pH 7, at 40 °C. [b] Δ*G*°=‐R*T*ln*K*_a_ and ‐*T*Δ*S*°=Δ*G*°–Δ*H*°. [c] *Q3‐sbl* sequence: 5’‐TTAGGGTGGTAGGGTGGGGAAGG‐3’.

Low‐affinity binding sites likely include the duplex domain and in particular the exposed 5’‐face of the quadruplex that has been found to be a favored binding site for indoloquinolines in a regular parallel *c‐Myc* quadruplex with its two exposed outer tetrads.[^22^, ^25^] For a direct comparison of binding affinities towards the Q‐D junction and a more exposed outer G‐tetrad, an additional quadruplex *Q3‐sbl* was introduced. The sequence of the latter closely resembles a *c‐Myc* variant that was reported to fold into a parallel quadruplex with a 4‐nt snapback loop and a 3’‐terminal G filling a vacant site of its 3’‐tetrad.[^30^, ^31^] With an additional mutation to match the 5’‐overhang in the *QD3‐sbl* hybrid, *Q3‐sbl* is expected to mimic *QD3‐sbl* lacking a Q‐D junction at its 3’‐outer tetrad. As an additional benefit, the relatively short diagonal snapback loop was previously shown to effectively prevent ligand binding, allowing better defined interactions only at the 5’‐face of *Q3‐sbl*.^[31]^

Initially, the anticipated snapback‐driven parallel fold of *Q3‐sbl* was demonstrated by NMR experiments (Figure S17). In line with a strong preference for the 5’‐tetrad, subsequent ITC titrations of *Q3‐sbl* with SYUIQ‐5 yielded a stoichiometry of 1 for high‐affinity binding. On the other hand, a corresponding association constant *K*_a_ ∼2 ⋅ 10^6^ M^−1^ was smaller by a factor of five compared to binding at the Q‐D interface in *QD3‐sbl*, identifying the Q‐D junction as a superior binding site for the indoloquinoline ligand (Table 1). Notably, the heat initially released upon ligand binding at the 5’‐outer tetrad of *Q3‐sbl* matches the first plateau region that follows the high‐affinity binding of SYUIQ‐5 to *QD3‐sbl*. This suggests a first binding event at the junction with subsequent binding at the 5’‐face and possibly additional binding at the duplex domain of the Q‐D hybrid.

## Discussion

Indoloquinoline ring systems feature a shape that maximizes π–π stacking interactions through intercalation between two guanine bases of the outer tetrad and the adjacent CG base pair in a Q‐D hybrid. In addition, electrostatic interactions are promoted by the positive potential at the *N*5‐protonated or *N*5‐methylated quinoline nitrogen. The specific ligand alignment allows them to be directed towards the central channel of the G‐core lined with the G carbonyl oxygen atoms but also towards the carbonyl oxygen of the GC base pair on top. The significance of the latter on binding may only be moderate. Conspicuously, however, cryptolepine was reported to feature a rather peculiar preference for intercalating between two CG base pairs when binding a B‐type DNA duplex. In a corresponding crystal structure, stacking interactions were optimized by the excellent geometric fit of cryptolepine with the neighboring CG base pairs.^[32]^ Notably, in close correspondence with the present SYUIQ‐5 complex structure, orientation of cryptolepine in the intercalation pocket positioned the quinoline portion of the ligand between the two guanines, allowing a close contact of the positively charged cryptolepine *N*5‐methyl to both of the 6‐carbonyl oxygens of the two stacked Watson‐Crick paired guanines in the duplex major groove.

Disregarding any significant steric or electronic effects due to *N*5‐methylation, the sidechain appended to the tetracyclic ring system in SYUIQ‐5 provides for additional binding affinity through its interactions at or within the grooves, considerably increasing affinity constants when compared to cryptolepine. Also, NMR data hint at cryptolepine being subjected to enhanced exchange processes between different ligand orientations. A 180° ring flip of the indoloquinoline possibly followed by minor translational adjustments is easily conceivable for cryptolepine but clearly hampered by the SYUIQ‐5 sidechain interacting within a groove. Thus, SYUIQ‐5 may be restricted to bind in a more defined orientation.

The only two high‐resolution structures reported to date for Q‐D hybrids complexed with ligands have revealed two rather divergent binding modes. One study reported on the binding of simple mono‐ and polyaromatic compounds built on a benzylamine substructure to the *QD2‐l* antiparallel quadruplex with its central hairpin‐type lateral loop.^[33]^ A bis‐aminomethylated anthracene ligand stacks on the two exposed guanines of the outer G‐tetrad at the junction, being in‐plane with the interfacial first GC base pair to form a pseudo‐triad. One of the two protonated, positively charged amino substituents on the ligand points towards the central channel of the G‐core. In addition to interactions with the central electron‐rich guanine oxygen atoms, weaker electrostatic and hydrogen bond interactions with opposite residues at the duplex major groove can also be envisaged. Conspicuously, such a binding geometry is reminiscent of several complexes with polycyclic ligands stacking on a quadruplex outer tetrad. In contrast to macrocycles covering the whole tetrad area, these ligands are often found to bind opposite of an in‐plane base recruited from overhang but also loop sequences.[^22^, ^34^, ^35^] Possible hydrogen bond interactions within such a pseudo‐base pair stacked on top of the outer G‐tetrad are often supplemented with a loose cap of another overhang/loop residue.

In the same study, some of the ligands were also used to bind the major G‐quadruplex formed in the U3 promoter region of the HIV‐1 long terminal repeat (*LTR‐III*), being of considerable interest as a novel antiviral target. Notably, the *LTR‐III* quadruplex comprises a 12‐nt diagonal loop with a duplex‐stem but with highly dynamic residues between quadruplex and duplex domains.^[4]^ Although no three‐dimensional complex structure has been reported, biophysical data suggested analogous binding modes for this class of ligands when targeting *QD2‐l* and biologically relevant *LTR‐III*.^[33]^

Another high‐resolution structure with binding at the Q‐D junction was determined for a conjugated ligand composed of a quadruplex‐specific naphthalene diimide (NDI) core linked to a positively charged platinum coordination complex [Pt(dien)(py)].^[36]^ In close correspondence to the present indoloquinoline binding, the NDI ring system was found to be sandwiched between interfacial outer tetrad and neighboring base pair of a quadruplex with a lateral duplex stem‐loop. Specific binding was further promoted by the platinum coordinated sidechain, interacting within the duplex minor groove through hydrogen bonds and electrostatic interactions.

It should be pointed out that the potential intercalation of a ligand between two G‐tetrads should likewise yield favorable binding energies through π–π stacking interactions with two adjacent tetrads. However, such a binding mode has not yet been confirmed on short cation‐stabilized quadruplexes and only intercalation between non‐conventional GAGA and GCGC quartets of an unusual G‐rich tetrahelical structure has been reported for a bis‐quinolinium compound.^[37]^ Also, porphyrin intercalation into long G4 DNA nanowires has only been evidenced in the absence of monovalent cations whereas non‐intercalative binding was suggested in a K^+^ solution.^[38]^ Apparently, in addition to the considerable energetic cost when unstacking G‐tetrads associated with the unwinding of four strands to provide for an intercalation pocket, a metal ion located within the central channel between tetrads seems to restrict access of a corresponding ligand.

**Figure 9:**
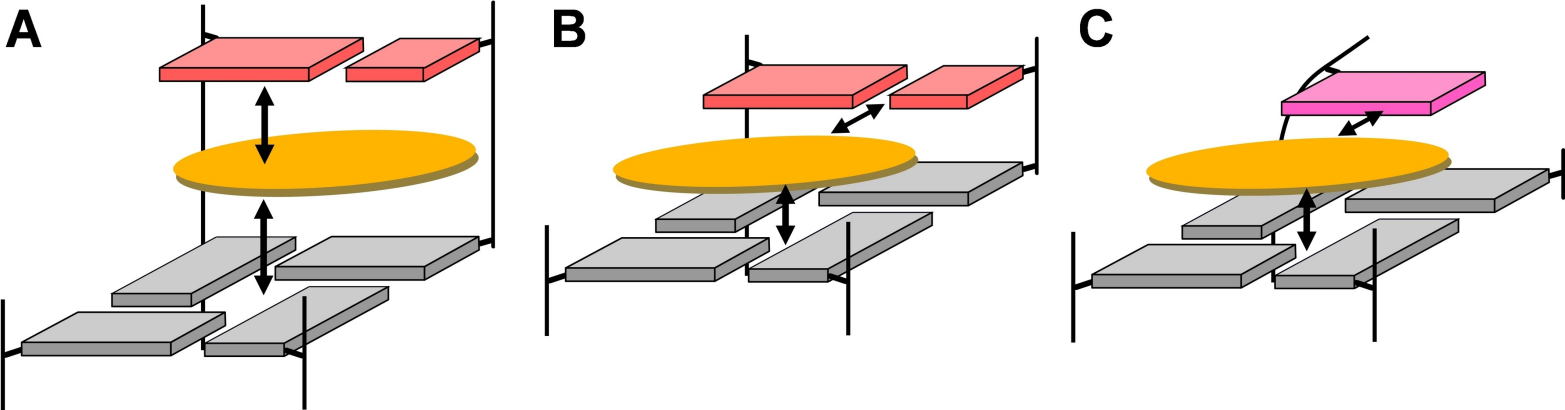
Schematic representation of binding modes for a ligand aromatic moiety to a quadruplex outer tetrad. (A) Intercalation between G‐tetrad and base pair at a Q‐D junction. (B) Stacking on outer tetrad in‐plane with an interfacial base pair at the junction. (C) Stacking on outer tetrad in‐plane with an overhang or loop residue of an isolated quadruplex without Q‐D interface. Arrows indicate direction of major π–π stacking, electrostatic, and/or hydrogen bond interactions of the orange‐colored ligand; bases of the G‐tetrad, base pair, and overhang or loop are colored gray, red, and magenta, respectively.

Taken together, planar aromatic ring systems with surface areas only covering part of a G‐tetrad as mostly found for quadruplex ligands may bind in two distinctive modes at a Q‐D junction (Figure 9). Both involve vertical π–π stacking onto the outer tetrad as a major contributor to binding. Additional horizontal electrostatic and hydrogen bond interactions with the interfacial base pair add to the complex stabilization in case of the base pair aligned opposite and in‐plane with the ligand. On the other hand, intercalation between outer tetrad and a duplex base pair at a Q‐D junction may occur if vertical stacking and electrostatic interactions of the intercalated ligand aromatic moiety overcome the energetic penalty associated with unwinding at the interface to create a binding pocket. It can be assumed that a matched shape and electrostatic potential of ligand and intercalation pocket will strongly favor ligand insertion. In this context it is worth mentioning that a Q‐D hybrid structure featuring a base triad platform between quadruplex and duplex motifs in its crystal form was unable to bind a ligand, most likely as a result of a largely occluded G‐tetrad surface area in case of an in‐plane binding mode or of a larger energy barrier for strand unwinding at the tetrad‐triad junction in case of an intercalative binding mode.^[39]^

Although being highly flexible in many cases, ligand aliphatic sidechains are important in providing for additional short‐lived electrostatic, hydrogen bond, and/or van der Waals interactions. In case of an intercalative binding mode, sidechains seem to favor interactions within the minor groove of the duplex domain and if appropriate may not only increase affinities but also selectivities towards the target hybrid. Finally, as suggested by the NMR analysis of unsubstituted cryptolepine, sidechains may effectively restrict ligand dynamics and exchange between different ligand alignments to fix a major ligand orientation.

Interest in structural details of Q‐D interfaces and in their recognition by ligands increasingly grow with the realization that various Q‐D junctions can potentially form within G‐rich sequences of genomic DNA. It seems obvious to utilize the unique features of quadruplex‐duplex interfaces for various technological applications, for example as additional structural motifs in aptamer constructs, but also as hotspots for drug targeting, trying to improve affinities and especially selectivities towards a particular quadruplex‐forming site. Up to now, the design of selective and potent ligands to target Q‐D junctions is an area still in its very infancy. An obvious approach based on the combination of a large aromatic surface area of a G‐selective ligand with typical duplex minor groove binders may suffer from the large size and molecular weight of the conjugates. On the other hand, the specific targeting of Q‐D junctions with small molecules needs more systematic studies. The three‐dimensional structure of a Q‐D junction complexed with indoloquinolines adds valuable information on the binding selectivity and the ligand binding mode. Critical interactions seem to rely on structural but also electrostatic complementarity that may be strengthened through additional hydrogen bond interactions, for example by appropriate sidechains. The results presented may thus constitute a helpful guide for the future design and development of ligands specifically targeting Q‐D interfaces.

## Experimental Section

### Materials and sample preparation

DNA oligonucleotides were synthesized by TIBMOLBIOL (Berlin, Germany). Samples were additionally purified by ethanol precipitation. Concentration of oligonucleotides was determined by their absorbance A_260_ at 80 °C using a molar extinction coefficient as provided by the manufacturer. The concentration of commercially available SYUIQ‐5 and cryptolepine (Sigma‐Aldrich Chemie GmbH, Germany) was determined from its weighed mass. Except for the ITC experiments, samples were dissolved in 10 mM potassium phosphate buffer, pH 7.0.

### UV melting experiments

UV melting experiments were performed with a Jasco V‐650 spectrophotometer (Jasco, Tokyo, Japan) equipped with a Peltier thermostat. For duplex melting, the hyperchromicity of the oligonucleotide solution (2 μM) was followed at λ=260 nm as a function of temperature. For melting of the quadruplex domain, the hypochromicity of the oligonucleotide solution (5 μM) was observed at λ=295 nm. Data were recorded from 10 °C to 90 °C with a heating rate of 0.2 °C min^−1^ and a bandwidth of 1 nm. Melting temperatures were determined by the first derivative of the melting curve. Melting temperatures of the DNA‐indoloquinoline complexes were not determined due to their broad, uncooperative melting profiles. All experiments were done in triplicate.

### Differential scanning calorimetry (DSC)

To circumvent inaccuracies in UV melting due to mutual perturbations of duplex and quadruplex absorbances, melting temperatures for a *QD3‐sbl* solution (50 μM) were additionally determined by DSC. DSC experiments were performed with a VP‐DSC instrument (Malvern Instruments, United Kingdom). The sample was heated with a heating rate of 0.5 °C min^−1^ from 10 °C to 80 °C. Melting temperatures and enthalpy values were determined from a second sample vs. buffer scan after subtracting a buffer vs. buffer scan. A cubic baseline fitting was used and the two peaks associated with duplex and quadruplex melting were deconvoluted. Data were analyzed with the Origin software.

### CD spectroscopy

CD spectra were recorded at 20 °C with a Jasco J‐810 spectropolarimeter equipped with a Peltier thermostat (Jasco, Tokyo, Japan). For recording CD spectra of the Q‐D hybrid (5 μM), a bandwidth of 1 nm, a scanning speed of 50 nm min^−1^, a response time of 4 s, and 5 accumulations were used. A concentrated solution of SYUIQ‐5 in DMSO was used for titrations up to a 5 : 1 ligand‐to‐DNA molar ratio. All spectra were blank‐corrected.

### NMR spectroscopy

For NMR experiments, a Bruker Avance 600 MHz NMR spectrometer equipped with an inverse ^1^H/^13^C/^15^N/^19^F quadruple resonance cryoprobehead and z‐field gradients was used. Spectra were processed in TopSpin 4.0.7 and assigned in CcpNMR V2.^[40]^ Unless indicated otherwise, spectra were acquired on samples in 90 % H_2_O/10 % D_2_O buffered with 10 mM potassium phosphate, pH 7.0. SYUIQ‐5 was used as a concentrated stock solution in DMSO‐d_6_. The final concentration of DMSO after the addition of one equivalent of ligand was about 2 %. Proton chemical shifts were referenced to TSP through the temperature dependent water chemical shift while ^13^C chemical shifts were referenced to DSS through an indirect referencing method. For solvent suppression, a WATERGATE w5 pulse scheme was employed in 1D and 2D NOESY experiments whereas a 3–9–19 water suppression scheme was used for DQF‐COSY, TOCSY, and ^1^H‐^13^C HSQC experiments. ^1^H‐^13^C HSQC spectra were acquired with 4 K×500 data points, a 1 s recycle delay, and a spectral width of 7500 Hz in the F1 dimension to cover aromatic C8/C6/C2 carbon resonances of the nucleobases. DQF‐COSY and TOCSY spectra with a mixing time of 80 ms and a DIPSI‐2 isotropic mixing scheme were recorded with 4 K×500 data points. 2D NOESY spectra with 80, 150, and 300 ms mixing times and EASY‐ROESY spectra acquired with a 80 ms mixing time and a 50° spinlock angle were acquired with 2 K×1 K data points. For all 2D homonuclear experiments a 2 s recycle delay was used. Spectra were zero‐filled to 4 K×1 K data points and processed with a squared sine‐bell window function except for 1D experiments which were multiplied with an exponential function.

### ITC experiments

ITC experiments were performed with a Microcal PEAQ‐ITC microcalorimeter (Malvern Instruments, United Kingdom) employing a reference power of 4 μcal s^−1^. Oligonucleotides and indoloquinoline ligands were dissolved in 20 mM potassium phosphate buffer, pH 7.0, supplemented with 100 mM KCl and 5 % DMSO. A ligand solution (1.5 μL, 400 μM) was titrated to the oligonucleotide solution (20 μM) with an injection duration of 3 s and a spacing of 240 s. The first injection (0.4 μL) was discarded before data analysis. Thermograms were subsequently fitted to a model with two sets of binding sites with the Microcal‐PEAQ ITC analysis software. All experiments were blank‐ and concentration‐corrected and performed in triplicate.

### Structure calculations

Employing NMR‐derived distance and dihedral angle restraints as well as H‐bond, planarity and repulsion restraints, 100 starting structures were generated for both free and complexed DNA by a simulated annealing protocol in XPLOR‐NIH 3.0.3.^[41]^ Structures were refined using AMBER16 with the parmbsc force field and OL15 modifications for DNA. An additional force field was employed for the ligand and parameterized for AMBER using the R.E.D server.^[42]^ Geometry optimization for the ligand was done with Hartree‐Fock calculations and a 6‐31G* basis set and the force field parameters were adapted from parm10 and GAFF. The 100 starting structures were subjected to a simulated annealing protocol to yield 20 lowest‐energy structures. Refinement in water was done by neutralizing the DNA with potassium ions, placing two ions in the inner core of the G‐quadruplex between two tetrad layers, and soaking the system with TIP3P water in a 10 Å truncated octahedral box. The final simulation was done at 1 atm and 300 K for 4 ns using only NOE‐ and hydrogen bond‐based distance restraints. For free *QD3‐sbl*, the trajectory was averaged for the last 500 ps. In contrast, only the last snapshot was used in the complex calculations to prevent distortions of the flexible ligand aliphatic sidechain. Structures were further minimized to obtain ten lowest‐energy structures. Structure parameters were extracted with the X3DNA web package.^[43]^ More details of the structure calculations are given in the Supporting Information.

## Conflict of interest

The authors declare no conflict of interest.

1

## Supporting information

As a service to our authors and readers, this journal provides supporting information supplied by the authors. Such materials are peer reviewed and may be re‐organized for online delivery, but are not copy‐edited or typeset. Technical support issues arising from supporting information (other than missing files) should be addressed to the authors.

Supporting Information

## Data Availability

The atomic coordinates and chemical shifts for free *QD3‐sbl* (PDB 7PNE; BMRB 34664) and of its complex with SYUIQ‐5 (PDB 7PNG; BMRB 34665) have been deposited in the Protein Data Bank.
