# Supplementary material for: Indoloquinoline Ligands Favor Intercalation at Quadruplex‐Duplex Interfaces
Source: Chemistry. 2022 Jan 5;28(7):e202103718. doi: 10.1002/chem.202103718 (PMC9303235; doi:10.1002/chem.202103718)
Supplement: Supplementary file 1 — Supporting Information [file CHEM-28-0-s001.pdf]

Chemistry–A European Journal

Supporting Information

Indoloquinoline Ligands Favor Intercalation at Quadruplex-Duplex Interfaces

Yoanes Maria Vianney and Klaus Weisz*

Contents

Experimental details on the structure calculations S2

Table S1: Sequences of oligonucleotides S4

Figure S1: NOESY spectral regions of free *QD3-sbl* S4

Figure S2: ^{1}H-^{13}C HSQC spectral regions of free *QD3-sbl* S5

Figure S3: Determination of the sugar conformations in free *QD3-sbl* S6

Table S2: Compilation of ^{1}H and ^{13}C chemical shifts in free *QD3-sbl* S7

Table S3: NMR restraints and structural statistics S9

Figure S4: DSC melting curve of *QD3-sbl* S10

Table S4: Melting temperatures of *QD3-sbl* S10

Figure S5: CD titration of *QD3-sbl* with SYUIQ-5 S11

Figure S6: NOESY spectral regions of a *QD3-sbl* - SYUIQ-5 complex S12

Figure S7: ^{1}H-^{13}C HSQC spectral regions of a *QD3-sbl* - SYUIQ-5 complex S13

Figure S8: Determination of sugar conformations in a *QD3-sbl* - SYUIQ-5 complex S14

Figure S9: NOESY and ROESY spectra of free and SYUIQ-5-bound *QD3-sbl* S15

Figure S10: Proton resonance assignments of bound SYUIQ-5 S16

Table S5: Compilation of ^{1}H and ^{13}C chemical shifts in a *QD3-sbl* - SYUIQ-5 complex S17

Table S6: Compilation of ^{1}H chemical shifts in bound SYUIQ-5 S18

Table S7: List of intermolecular NOE contacts S19

Figure S11: NOESY spectral regions with intermolecular contacts S19

Figure S12: Q-D interface before and after ligand binding S20

Figure S13: NOESY spectral regions and topology of free *QD2-l* S21

Figure S14: NOESY spectral regions of a *QD3-sbl* – cryptolepine complex S22

Figure S15: NOESY and ROESY spectra of free and cryptolepine-bound *QD3-sbl* S23

Figure S16: ITC thermograms for ligand binding S24

Figure S17: NOESY spectral regions and topology of *Q3-sbl* S25

Structure calculations

For both free and complexed DNA, 100 starting structures were selected from 500 structures generated by a simulated annealing protocol in XPLOR-NIH 3.0.3.[1] Distance restraints were grouped based on cross-peak intensities in NOESY experiments. For free *QD3-sbl*, distances for non-exchangeable protons were fixed to 2.9 ± 1.1 Å for strong cross-peaks, 4.0 ± 1.5 Å for medium intensity cross-peaks, 5.5 ± 1.5 Å for weak cross-peaks, and 6.0 ± 1.5 Å for very weak cross-peaks. For exchangeable protons, distances were set to 2.9 ± 1.1 Å for strong cross-peaks, 4.0 ± 1.2 Å for medium intensity cross-peaks, 5.0 ± 1.2 Å for weak cross-peaks, and 6.0 ± 1.2 Å for very weak cross-peaks. A distance of 5.0 ± 2.0 Å was used for overlapped peaks. Distance ranges for the ligand-DNA complex were fixed according to distances of non-exchangeable protons in free *QD3-sbl* with no further discrimination between exchangeable and non-exchangeable protons due to broadening effects of exchangeable cross-peaks associated with higher uncertainties. Intermolecular distances between ligand and DNA protons were set to 4.0 +1.5/-2.5 Å for strong cross-peaks, 5.5 +1.5/-2.5 Å for weak cross-peaks, 6.0 +1.5/-2.5 Å for very weak cross-peaks, and 5.0 +2.0/-2.5 Å for overlapped peaks. Additional repulsion restraints were set to 10.0 ± 5.0 Å for non-exchangeable peaks. Torsion angles χ were set to *anti* for residues 1-35 (170° - 310°) and to *syn* for G36 (25° – 95°). All sugar puckers that could be determined based on DQF-COSY experiments were fixed to a south conformation with a pseudorotation angle of 144° – 180°. Empirical hydrogen bond-based distance and planarity restraints were added for the three G-tetrads and the seven Watson-Crick base pairs. For structure calculations of the complex, additional α (260° – 320°) and β (190° – 250°) backbone torsion angle restraints, adopted from experimental data also including dinucleotide-drug model complexes, were employed for residues C19 and G36.[2,3]

The structure was refined without planarity restraints for the duplex base-pairs using AMBER16 with the parmbsc force field and OL15 modifications for the DNA. For the complex, an additional force field was employed for the ligand and parameterized for AMBER using the R.E.D server.[4] Geometry optimization for the ligand was done with Hartree-Fock calculations and a 6-31G* basis set and the force field parameters were adapted from parm10 and GAFF. The 100 starting structures were subjected to a simulated annealing protocol to yield 20 lowest-energy structures. Restraint energies were 40 kcal·mol^{-1}·Å^{-2} for NOE-based distance restraints, 200 kcal·mol^{-1}·rad^{-2} for dihedral angle restraints, and 30 kcal·mol^{-1}·Å^{-2} for planarity restraints. The system was equilibrated at 300 K for 5 ps, followed by heating to 1000 K over 10 ps. Keeping this temperature for the next 30 ps, the system was subsequently cooled to 100 K within 45 ps and to 0 K within 10 ps.

Refinement in water was done by initially neutralizing the DNA with potassium ions, placing two ions in the inner core of the G-quadruplex between two tetrad layers. The system was soaked

with TIP3P water in a 10 Å truncated octahedral box and subsequently minimized with 500 steps of steepest descent minimization followed by another 500 steps of conjugate gradient minimization. The DNA was fixed with a force constant of 25 $kcal \cdot mol^{-1} \cdot Å^{-2}$. The system was heated up from 100 K to 300 K under constant volume in 10 ps and further equilibrated under a constant pressure of 1 atm with decreasing energy restraints of 5, 4, 3, 2, 1, and 0.5$\cdot kcal \cdot mol^{-1} \cdot Å^{-2}$. The final simulation was done at 1 atm and 300 K for 4 ns using only NOE- and hydrogen bond-based distance restraints. For free *QD3-sbl*, the trajectory was averaged for the last 500 ps. In contrast, only the last snapshot was used in the complex calculations to prevent distortions of the flexible ligand aliphatic sidechain. Structures were further minimized to obtain ten lowest-energy structures. Structure parameters were extracted with the X3DNA web package.[5]

References

[1] C. D. Schwieters, J. J. Kuszewski, G. Marius Clore, *Prog. Nucl. Magn. Reson. Spectrosc.* **2006**, *48*, 47–62.

[2] H.-S. Shieh, H. M. Berman, M. Dabrow, S. Neidle, *Nucleic Acids Res.* **1980**, *8*, 85–97.

[3] A. Adams, J. M. Guss, C. A. Collyer, W. A. Denny, L. P. G. Wakelin, *Biochemistry* **1999**, *38*, 9221–9233.

[4] E. Vanquelef, S. Simon, G. Marquant, E. Garcia, G. Klimerak, J. C. Delepine, P. Cieplak, F. Y. Dupradeau, *Nucleic Acids Res.* **2011**, *39*, 511–517.

[5] X. J. Lu, W. K. Olson, *Nucleic Acids Res.* **2003**, *31*, 5108–5121.

Table S1. Oligonucleotides used in this study.

name	sequence[a]
QD3-sbl	5'-TTAGGTGGGTAGGGTGGGCTAGTCATTTTGACTAGG-3'
QD2-l	5'-GGTTGGCGCGAAGCATTCGCGGGTTGG-3'
Q3-sbl	5'-TTAGGGTAGGTGGGTGGGGAAGG-3'

[a]G residues participating in G-tetrad formation are underlined.

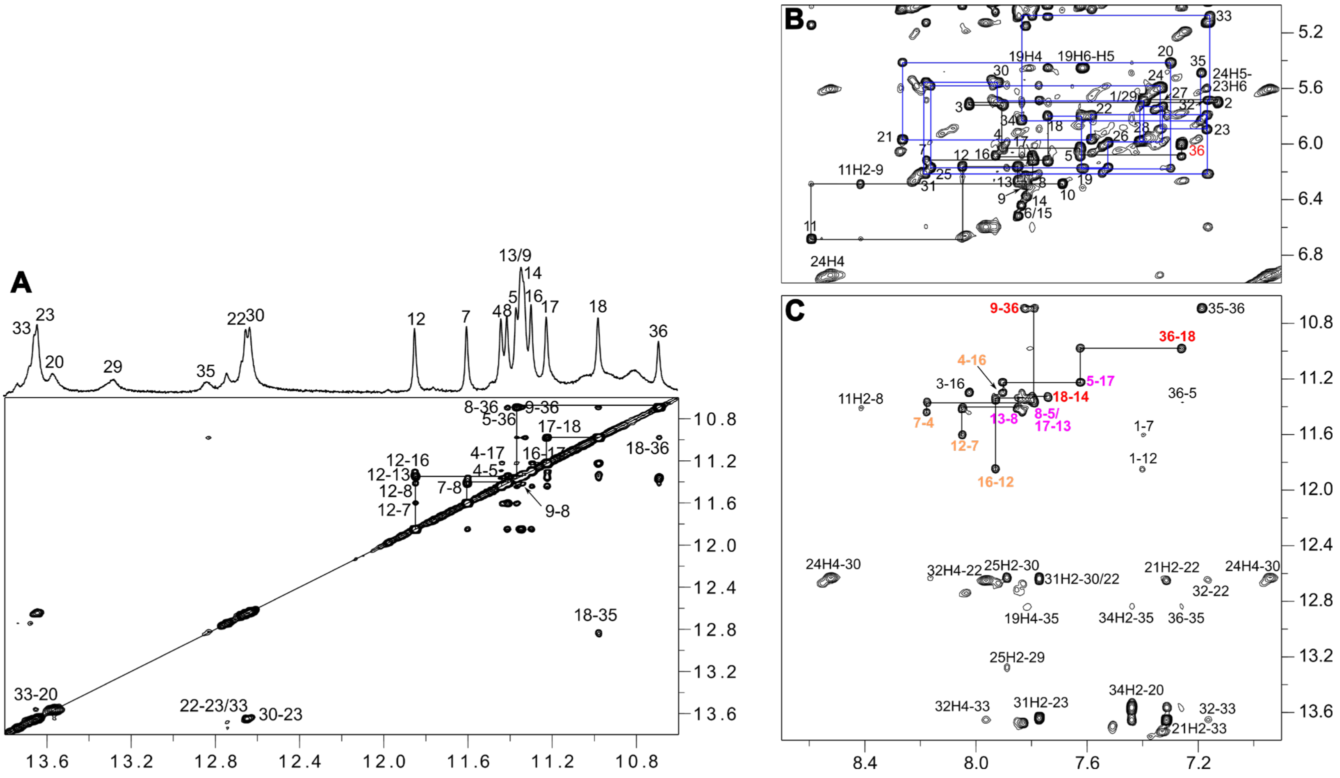

Figure S1. 2D NOESY spectrum (300 ms mixing time, 20 °C) of *QD3-sbl* (1 mM). (A) Imino(ω_2)-imino(ω_1) spectral region; a corresponding 1D spectrum with resonance assignments is given on top. (B) H8/6(ω_2)-H1'(ω_1) spectral region; an intra-nucleotide H8-H1' contact of *syn*-G36 is labeled in red and H8/H6-H1' connectivities within the duplex stem-loop are traced with blue lines. (C) H8/6/2(ω_2)-imino(ω_1) spectral region; intra-tetrad H8(ω_2)-H1(ω_1) cross-peaks are labeled with colors depending on G-tetrad layer; inter-tetrad connectivities along the G-columns are traced by horizontal and vertical lines.

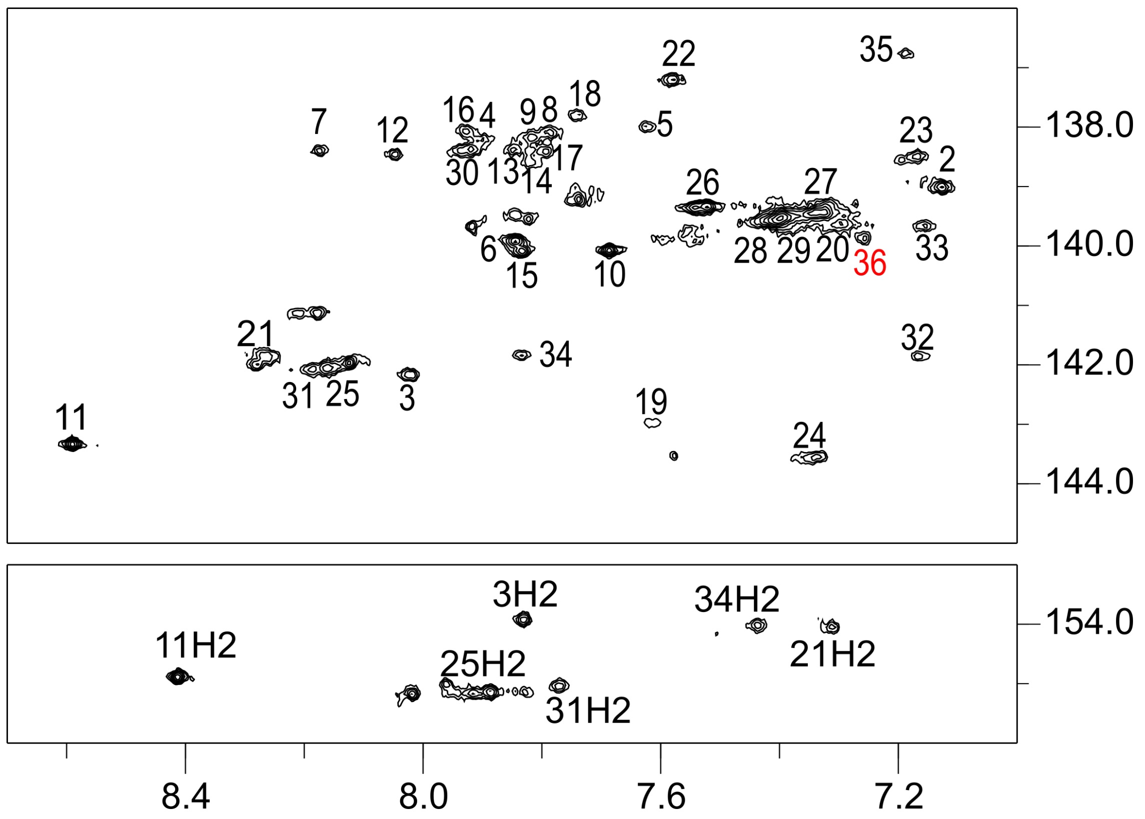

Figure S2. H8/H6(ω_2)-C8/C6(ω_1) (top) and H2(ω_2)-C2(ω_1) (bottom) spectral region of a ^{1}H-^{13}C HSQC spectrum of *QD3-sbl* (1 mM) acquired at 20 °C. The *syn*-G36 cross-peak is labeled in red.

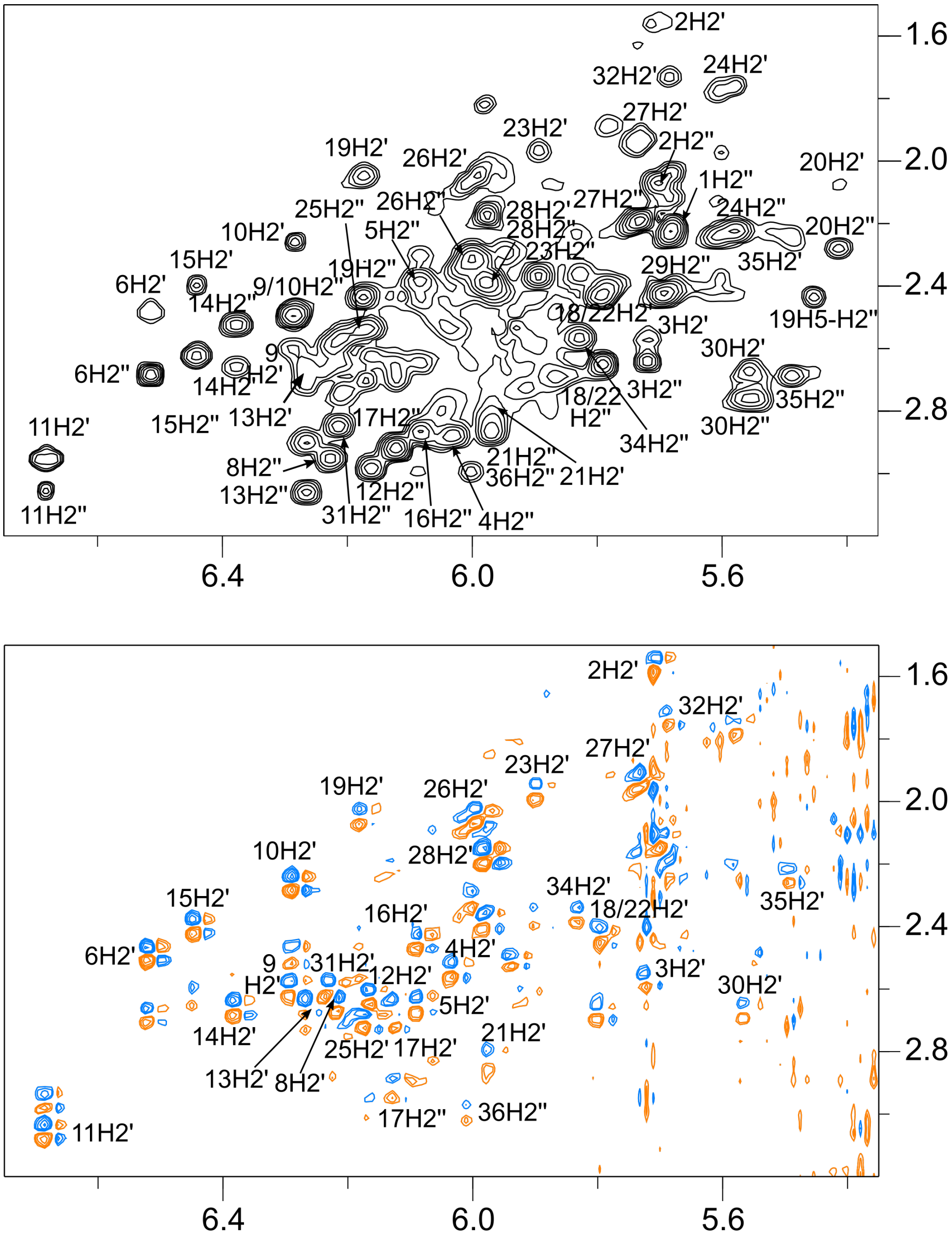

Figure S3. Determination of the sugar conformation. 2D NOESY (top, mixing time 80 ms) and DQF-COSY spectrum (bottom) with H1’(ω_2)-H2’/H2”(ω_1) cross-peaks of *QD3-sbl*.The spectra were acquired at 20 °C. Conformational analysis of DQF-COSY cross-peak patterns and scalar couplings followed stereospecific H2’/H2” assignments in the NOESY spectrum at short mixing times.

Table S2. ^{1}H and ^{13}C chemical shifts δ of *QD3-sbl.*[a]

δ (ppm)	H8/H6	H1/H3	H1’	H2’/H2”	H3’	H5/H2/Me	C8/C6	C2
T1	7.40	n.d	5.68	2.04/2.22	4.41	1.62	139.54	-
T2	7.13	n.d	5.70	1.57/2.07	4.51	1.50	139.02	-
A3	8.02	-	5.72	2.57/2.64	4.85	7.83	142.17	153.93
G4	7.90	11.44	6.03	2.54/2.88	4.91	-	138.22	-
G5	7.62	11.37	6.08	2.65/2.39	5.01	-	137.99	-
T6	7.85	n.d	6.51	2.48/2.68	5.08	1.99	139.93	-
G7	8.18	11.60	6.12	2.69/2.92	4.95	-	138.41	-
G8	7.79	11.41	6.22	2.60/2.96	5.01	-	138.07	-
G9	7.82	11.34	6.29	2.60/2.51	5.03	-	138.19	-
T10	7.69	n.d.	6.28	2.26/2.49	4.77	1.95	140.08	-
A11	8.59	-	6.68	3.06/2.96	5.15	8.41	143.34	154.91
G12	8.05	11.85	6.16	2.63/2.98	5.00	-	138.46	-
G13	7.85	11.35	6.27	2.65/3.06	5.04	-	138.39	-
G14	7.82	11.33	6.38	2.66/2.53	5.15	-	138.42	-
T15	7.84	n.d.	6.44	2.40/2.62	4.99	1.96	140.09	-
G16	7.93	11.30	6.08	2.45/2.87	5.05	-	138.09	-
G17	7.80	11.22	6.12	2.69/2.92	5.08	-	138.41	-
G18	7.74	10.98	5.79	2.43/2.65	5.00	-	137.82	-
C19	7.62	-	6.17	2.05/2.44	4.90	5.45	142.99	-
T20	7.30	13.56	5.41	2.08/2.28	4.85	1.62	139.63	-
A21	8.27	-	5.97	2.82/2.88	5.03	7.31	141.86	154.05
G22	7.58	12.65	5.79	2.43/2.65	4.79	-	137.21	-
T23	7.17	13.64	5.89	1.97/2.37	4.76	1.23	138.49	-
C24	7.34	-	5.58	1.77/2.23	4.76	5.61	143.57	-
A25	8.16	-	6.17	2.71/2.54	4.97	7.89	142.05	155.15

T26	7.52	n.d.	5.99	2.05/2.32	4.67	1.78	139.35	-
T27	7.33	n.d.	5.73	1.93/2.19	4.60	1.58	139.43	-
T28	7.41	n.d.	5.98	2.17/2.39	4.70	1.63	139.57	-
T29	7.39	13.28	5.69	2.13/2.43	4.70	1.82	139.53	-
G30	7.92	12.63	5.56	2.67/2.76	4.97	-	138.38	-
A31	8.18	-	6.21	2.65/2.85	4.99	7.77	142.06	155.04
C32	7.16	-	5.68	1.73/2.24	4.60	5.13	141.86	-
T33	7.16	13.65	5.08	1.84/1.94	4.60	1.43	139.67	-
A34	7.84	-	5.83	2.37/2.57	4.79	7.44	141.83	154.01
G35	7.19	12.84	5.49	2.24/2.69	4.86	-	136.77	-
G36	7.26	10.69	6.00	2.30/3.00	4.69	-	139.89	-

[a]At 20 ºC in 10 mM potassium phosphate buffer, pH 7.0.

Table S3. NMR restraints and structural statistics of calculated structures.

NOE distance restraints	*QD3-sbl*	*QD3-sbl* – SYUIQ-5
intra-residual	157	143
inter-residual	265	208
exchangeable	101	64
intermolecular	---	11
repulsion	---	3
other restraints:		
hydrogen bonds	82	82
dihedral angles	62	73
planarity	10	10
structural statistics:		
pairwise heavy atom RMSD value (Å)		
all residues	2.5 ± 0.43	2.75 ± 0.45
G-tetrad core	0.85 ± 0.24	1.00 ± 0.16
duplex stem-loop	1.80 ± 0.62	2.15 ± 0.64
NOE violations:		
maximum violation	0.275 Å	0.204 Å
number of violations (> 0.2)	0.1 ± 0.3	0.1 ± 0.3
mean NOE violation	0.002 ± 0.001	0.002 ± 0.0004
deviations from idealized geometry:		
bond lengths (Å)	0.01 ± 0.0001	0.01 ± 0.0001
bond angles (degree)	2.23 ± 0.03	2.35 ± 0.03

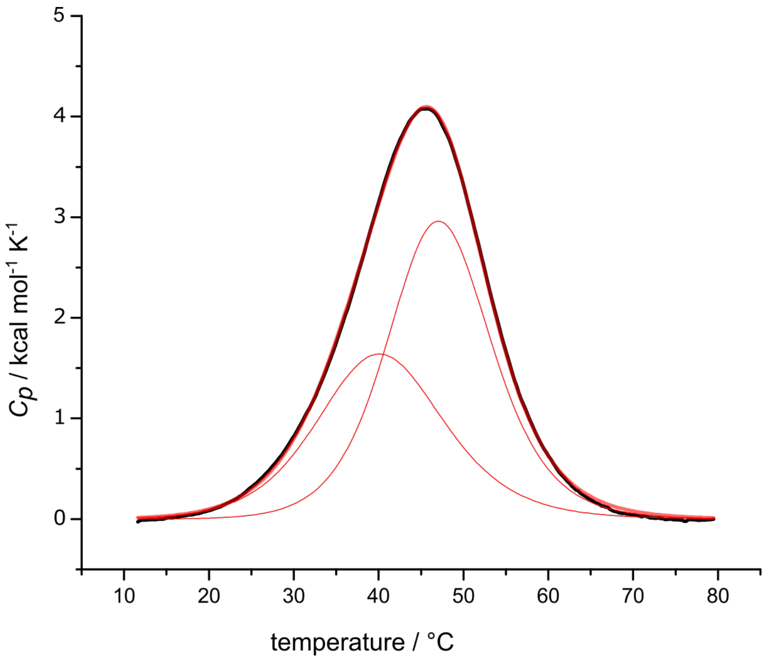

Figure S4. Representative DSC thermogram (black) for *QD3-sbl* in 10 mM KP_i buffer, pH 7.0, and superimposed fit with two melting transitions (red).

Table S4. Melting temperatures T_m of quadruplex and duplex domains of the *QD3-sbl* hybrid.

	T_m (°C)^a	T_m (°C)^b	$\Delta H°_{cal}$ (kcal/mol)^b	$\Delta H°_{vH}$ (kcal/mol)^b
quadruplex	40.5 ± 0.9	40.2 ± 0.5	33.3 ± 0.8	38.2 ± 1.2
duplex	47.5 ± 1.1	47.2 ± 0.2	49.1 ± 1.3	49.2 ± 0.6

[a]From UV melting; average values with standard deviations from three independent measurements.
[b]From DSC; average values with standard deviations from three independent measurements.

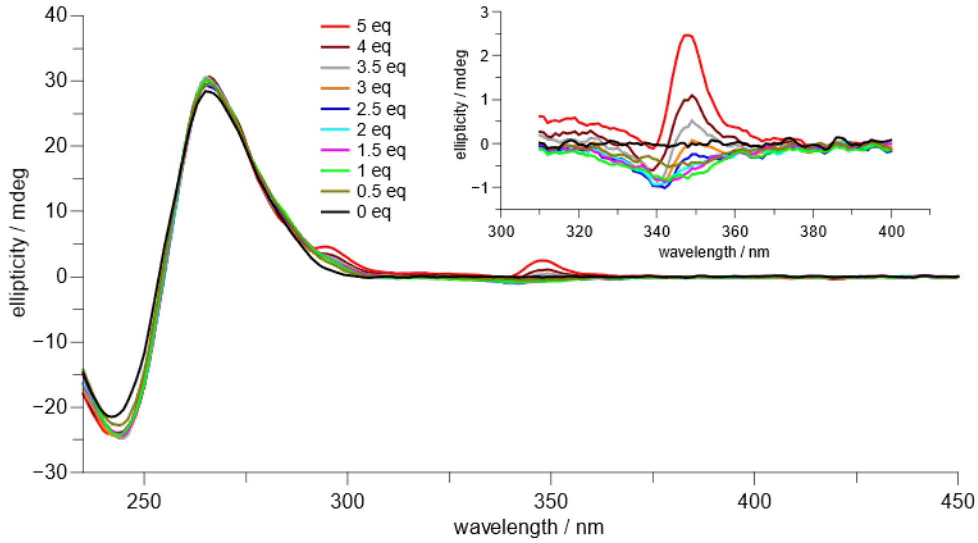

Figure S5. CD titration of *QD3-sbl* with SYUIQ-5 (0-5 equivalents). Spectra were acquired at 20 °C in 10 mM KP_i buffer, pH 7.0.

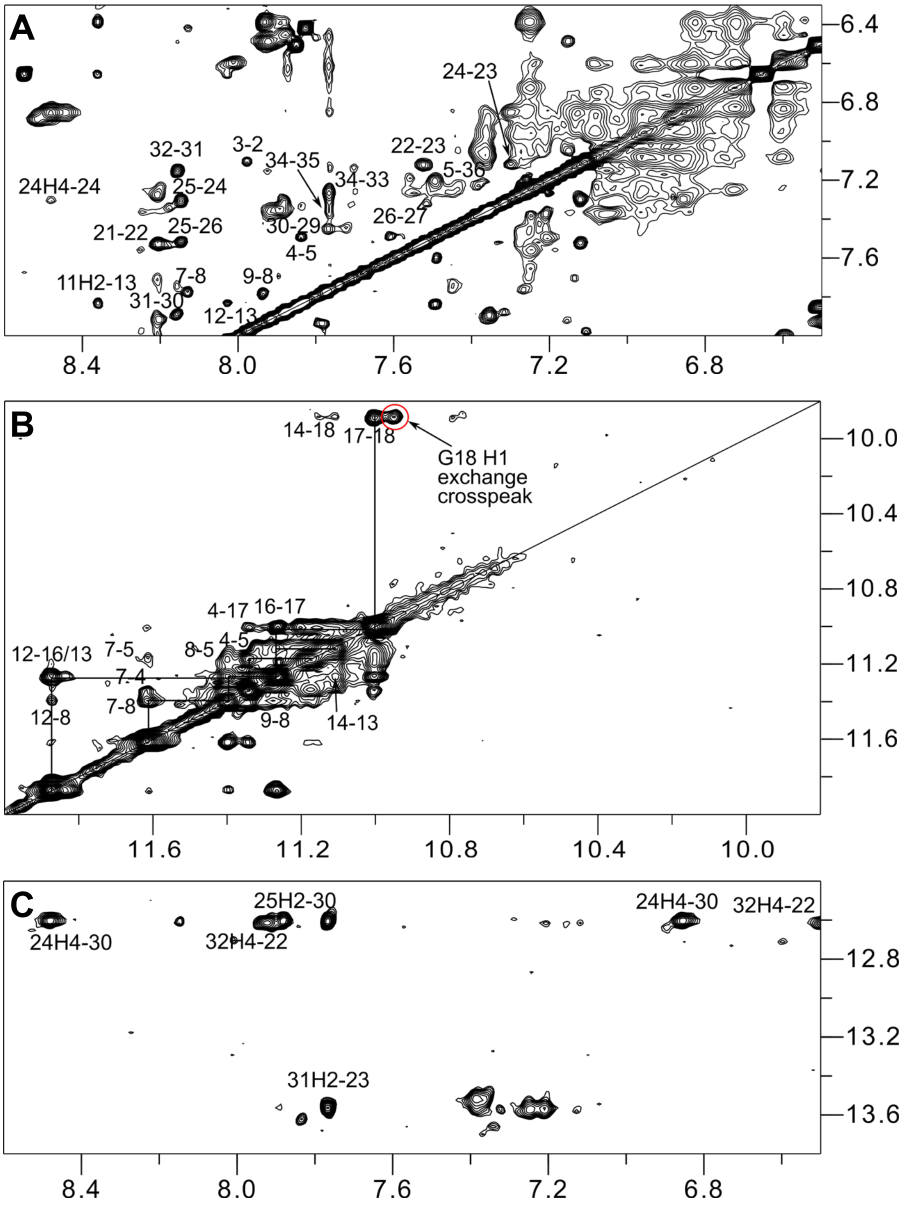

Figure S6. Regions of a 2D NOESY spectrum (300 ms mixing time, 30 °C) with assignments of non-exchangeable and exchangeable protons for a 1:1 mixture of SYUIQ-5 and *QD3-sbl* (1 mM). (A) H6/8(ω_2)-H6/8(ω_1), (B) H1(ω_2)-H1(ω_1), and (C) C H4/A H2(ω_2)-imino(ω_1) spectral region. In (B), an exchange cross-peak for G18 H1 between the free and the complexed structure is circled in red (for a corresponding ROESY spectrum see Figure S9).

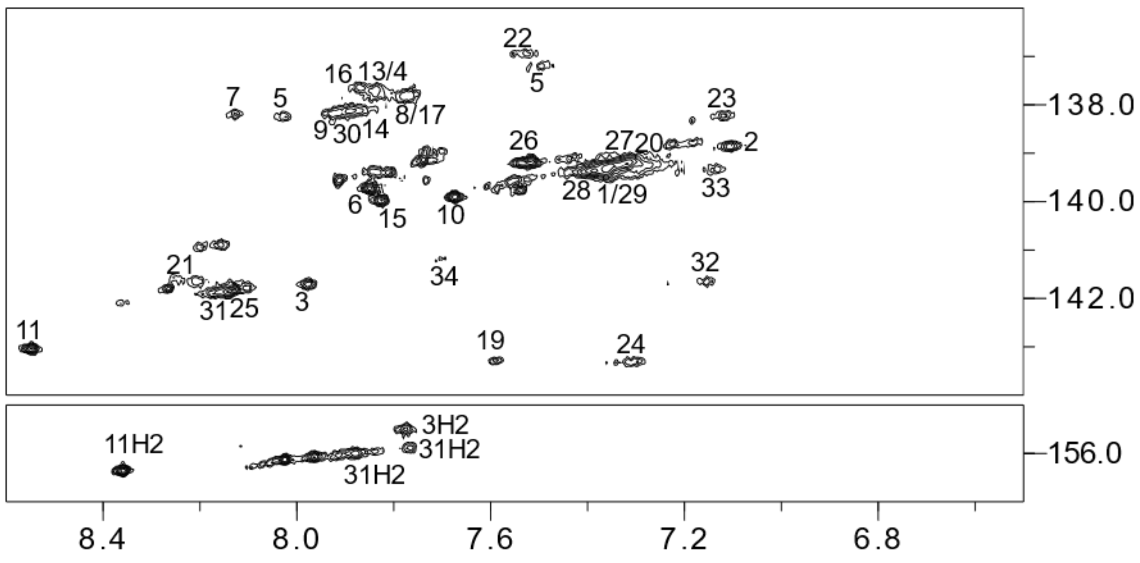

Figure S7. H8/H6(ω2)-C8/C6(ω1) (top) and H2(ω2)-C2(ω1) (bottom) HSQC spectral region of *QD3-sbl* (1 mM) in the presence of 1 eq. SYUIQ-5. The spectra were acquired at 30 °C.

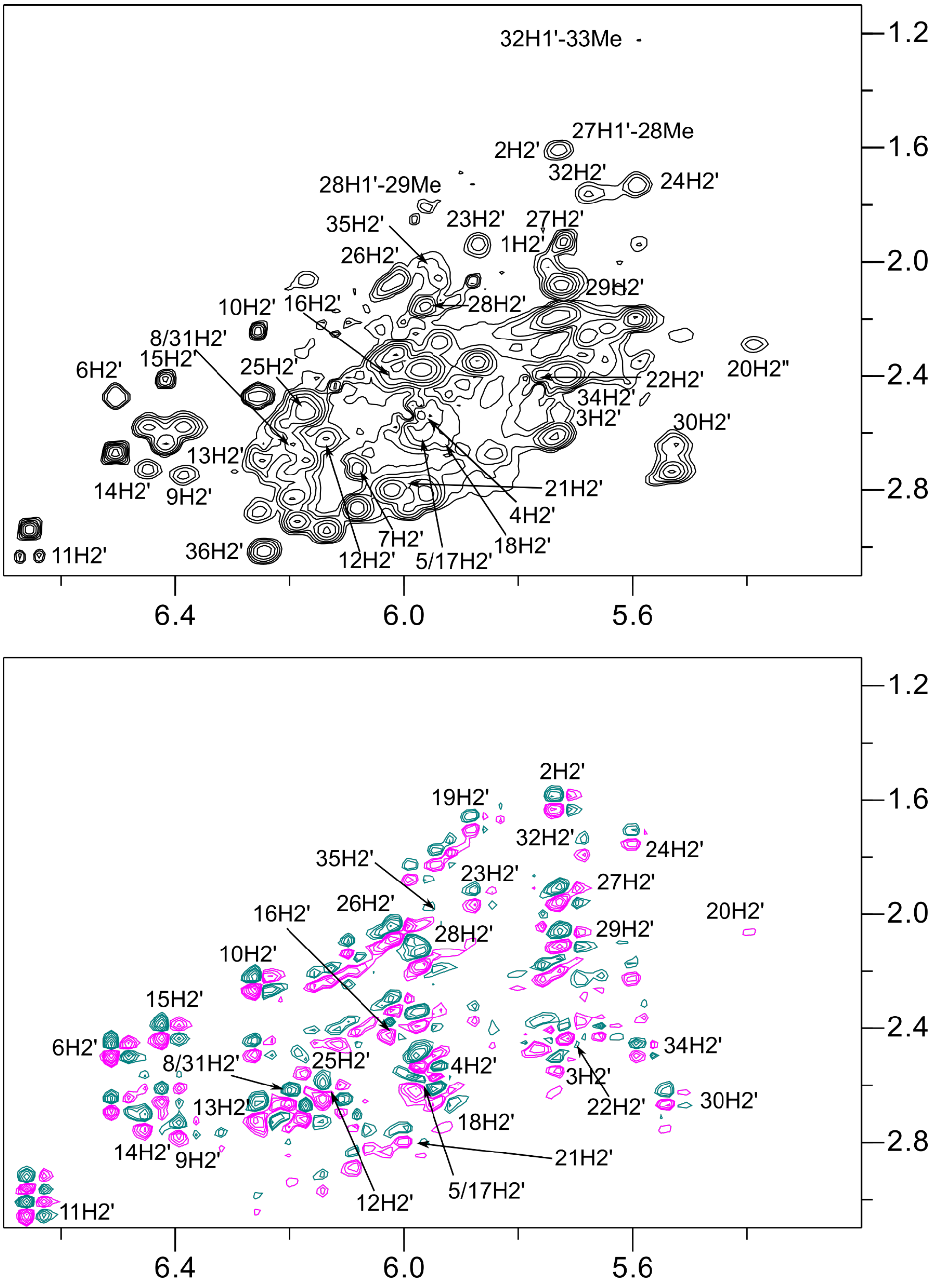

Figure S8. Determination of the sugar conformation in the 1:1 complex of *QD3-sbl* (1 mM) and SYUIQ-5. 2D NOESY (top, mixing time 80 ms) and DQF-COSY spectrum (bottom) with H1'(ω_2)-H2'/H2''(ω_1) cross-peaks. The spectra were acquired at 30 °C. Conformational analysis of DQF-COSY cross-peak patterns and scalar couplings followed stereospecific H2'/H2'' assignments in the NOESY spectrum at short mixing times; correlations for the T33 residue are only observed at lower threshold levels.

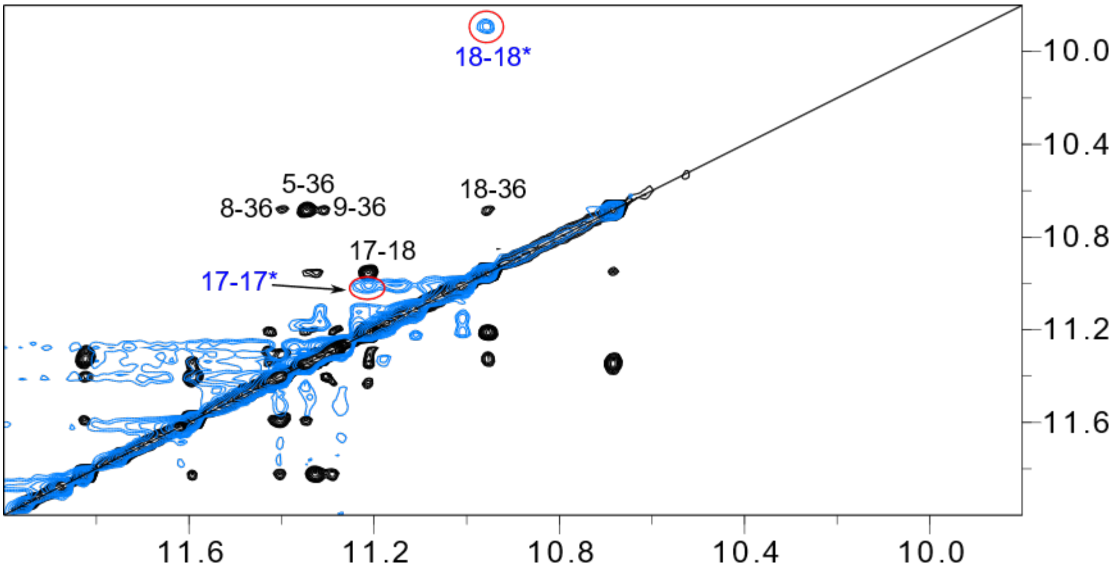

Figure S9. 2D NOESY spectrum of free *QD3-sbl* (300 ms mixing time, black) with superimposed ROESY spectrum of *QD3-sbl* in the presence of 0.5 eq. SYUIQ-5 (blue, only positive contours shown for clarity). Spectra show guanine H1(ω_2)-H1(ω_1) correlations of the G-core and were acquired at 30 °C. Exchange cross-peaks of G17 H1 and G18 H1 between free and complexed *QD3-sbl* are circled in red.

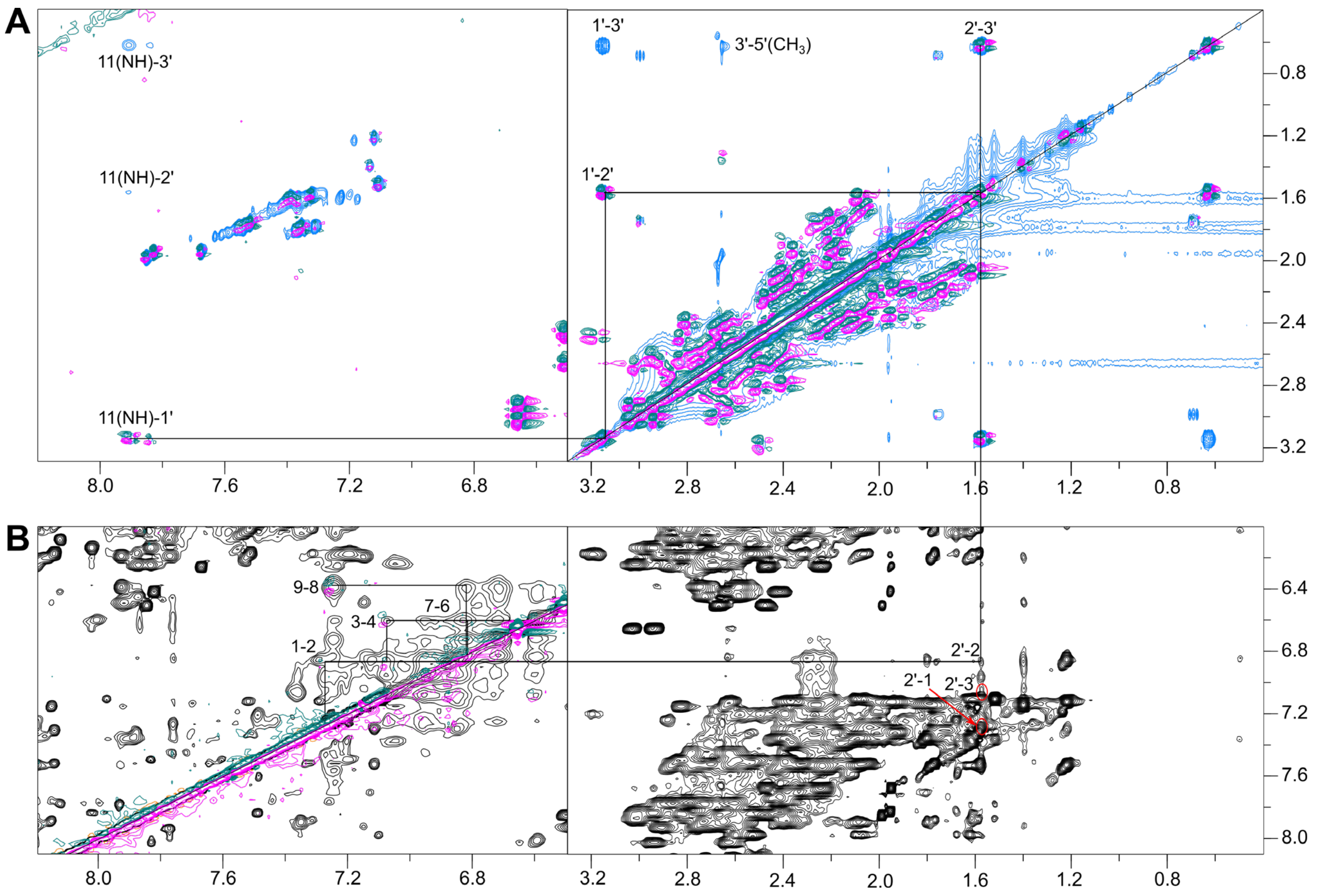

Figure S10. Proton resonance assignments of SYUIQ-5 bound to *QD3-sbl*. (A) Superimposed DQF-COSY and TOCSY spectra showing cross-peaks between SYUIQ-5 aliphatic side chain protons. (B) Superimposed DQF-COSY and NOESY spectra (300 ms mixing time) with correlations between aromatic protons (left) and NOESY spectral region with connectivities from SYUIQ-5 aliphatic side chain protons to aromatic protons of the quinoline subunit (right). Spectra were acquired at 30 °C.

Table S5. ^{1}H and ^{13}C chemical shifts δ of *QD3-sbl* complexed with SYUIQ-5 in a 1:1 molar ratio.[a]

δ (ppm)	H8/H6	H1/H3	H1’	H2’/H2”	H3’	H5/H2/Me	C8/C6	C2
T1	7.36	n.d	5.75	2.02/2.21	4.43	1.62	139.33	-
T2	7.11	n.d	5.73	1.61/2.08	4.51	1.52	138.87	-
A3	7.98	-	5.73	2.53/2.60	4.82	7.77	141.71	154.51
G4	7.84	11.34	5.96	2.51/2.83	4.90	-	137.73	-
G5	7.49	11.18	5.98	2.58/2.39	5.00	-	137.20	-
T6	7.85	n.d	6.50	2.48/2.67	5.08	1.99	139.72	-
G7	8.13	11.61	6.08	2.73/2.86	4.87	-	138.21	-
G8	7.78	11.40	6.19	2.64/2.91	5.02	-	137.83	-
G9	7.93	11.27	6.39	2.75/2.58	5.04	-	138.17	-
T10	7.68	n.d.	6.26	2.24/2.47	4.76	1.95	139.91	-
A11	8.55	-	6.65	3.03/2.94	5.14	8.36	143.06	155.36
G12	8.03	11.87	6.13	2.62/2.94	4.99	-	138.25	-
G13	7.84	11.26	6.24	2.69/3.02	5.03	-	137.73	-
G14	7.88	11.11	6.45	2.73/2.58	5.15	-	138.12	-
T15	7.83	n.d.	6.42	2.41/2.63	5.01	1.95	139.98	-
G16	7.87	11.26	6.02	2.40/2.80	5.03	-	137.63	-
G17	7.77	11.00	5.96	2.60/2.81	5.05	-	137.82	-
G18	7.45	9.89	5.93	2.63/2.78	n.d.	-	n.d.	-
C19	7.59	-	5.87	1.68/n.d.	n.d.	5.72	143.31	-
T20	7.28	n.d.	5.39	2.04/2.29	4.80	1.58	139.26	-
A21	8.21	-	5.99	2.77/2.87	5.00	n.d.	141.66	n.d.
G22	7.53	12.61	5.74	2.39/2.62	4.78	-	136.93	-
T23	7.12	13.56	5.87	1.94/2.35	4.75	1.22	138.24	-
C24	7.30	-	5.59	1.73/2.19	4.74	5.59	143.31	-
A25	8.15	-	6.17	2.70/2.53	4.96	7.88	141.86	155.00

T26	7.52	n.d.	6.01	2.07/2.32	4.67	1.77	139.18	-
T27	7.32	n.d.	5.72	1.93/2.18	4.59	1.59	139.22	-
T28	7.39	n.d.	5.96	2.16/2.37	4.69	1.61	139.37	-
T29	7.35	n.d.	5.72	2.08/2.40	4.68	1.80	139.28	-
G30	7.89	12.60	5.53	2.64/2.73	4.95	-	138.12	-
A31	8.15	-	6.19	2.64/2.83	4.96	7.77	141.87	154.89
C32	7.15	-	5.68	1.77/2.26	4.60	5.11	141.65	-
T33	7.14	n.d.	5.22	1.80/1.99	4.63	1.40	139.34	-
A34	7.70	-	5.64	2.41/2.34	4.78	n.d.	141.19	n.d.
G35	7.19	n.d.	5.95	2.01/2.40	n.d.	-	n.d.	-
G36	7.20	n.d.	6.17	2.26./3.19	n.d.	-	n.d.	-

[a]At 30 ºC in 10 mM potassium phosphate buffer, pH 7.0.

Table S6. ^{1}H chemical shifts of SYUIQ-5 bound to *QD3-sbl* at a 1:1 molar ratio.[a]

δ (ppm)	LH1	LH2	LH3	LH4	LNH5	LH6	LH7	LH8
	7.28	6.88	7.08	6.60	n.d.	6.60	6.83	6.38
δ (ppm)	LH9	LNH10	LNH11	LH1’	LH2’	LH3’	LH5’	
	7.25	n.d.	7.90	3.15	1.57	0.62	2.66	

[a]At 30 ºC in 10 mM potassium phosphate buffer, pH 7.0.

Table S7. Intermolecular NOE contacts observed between *QD3-sbl* and bound SYUIQ-5.

proton		cross-peak intensity			
ligand	Q-D hybrid	strong	weak	very weak	overlapped
LH1	G35 H2’				
LH4	G9 H1				
LH6	G9 H1				
LH7	G14 H1’				
LH7	G18 H2’				
LH7	G18 H8				
LH8	C19 H6				
LH9	G14 H1				
LH9	G18 H1				
LH9	G18 H8				
LH2’a/b	G5 H2”				

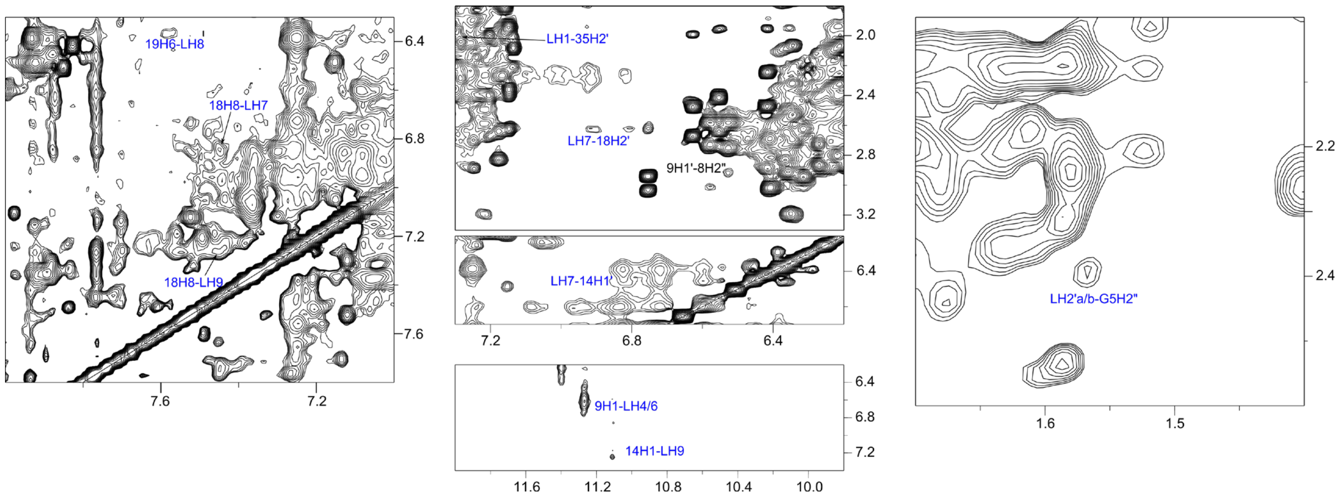

Figure S11. Expanded spectral regions of a 2D NOESY spectrum (300 ms mixing time, 30 °C) on a *QD3-sbl* - SYUIQ-5 1:1 mixture with intermolecular NOE contacts between ligand and the Q-D hybrid labeled in blue.

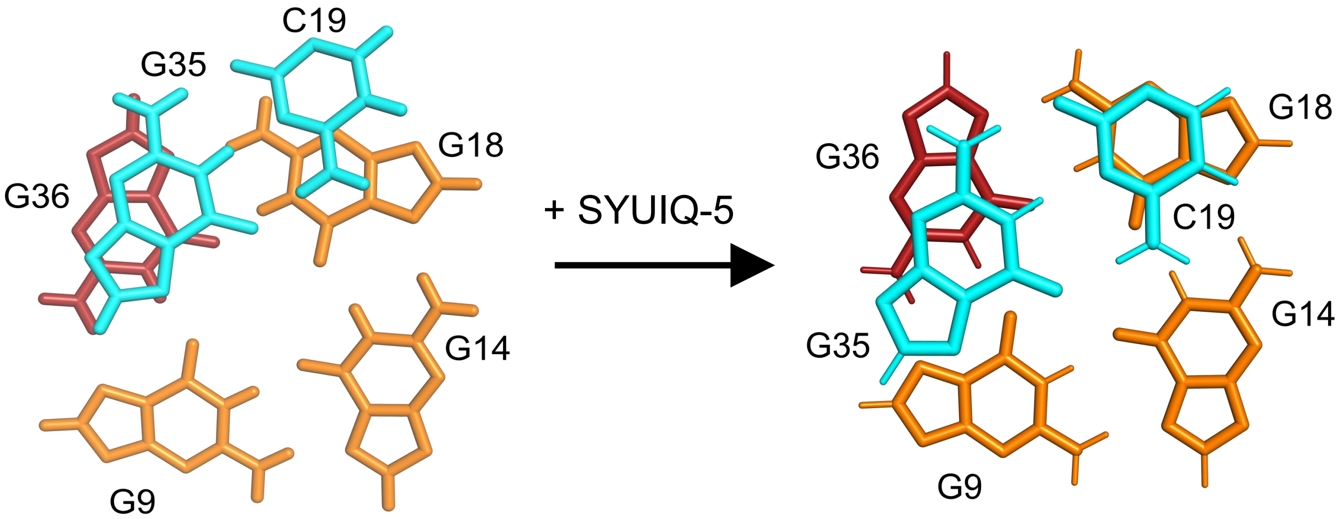

Figure S12. Q-D interface with 3'-outer tetrad and interfacial CG base pair on top. The latter is shifted towards the center of the G-core after ligand binding (ligand has been omitted for clarity); *anti*-G residues of the tetrad, *syn*-G36, and the CG base pair are colored orange, red, and cyan, respectively.

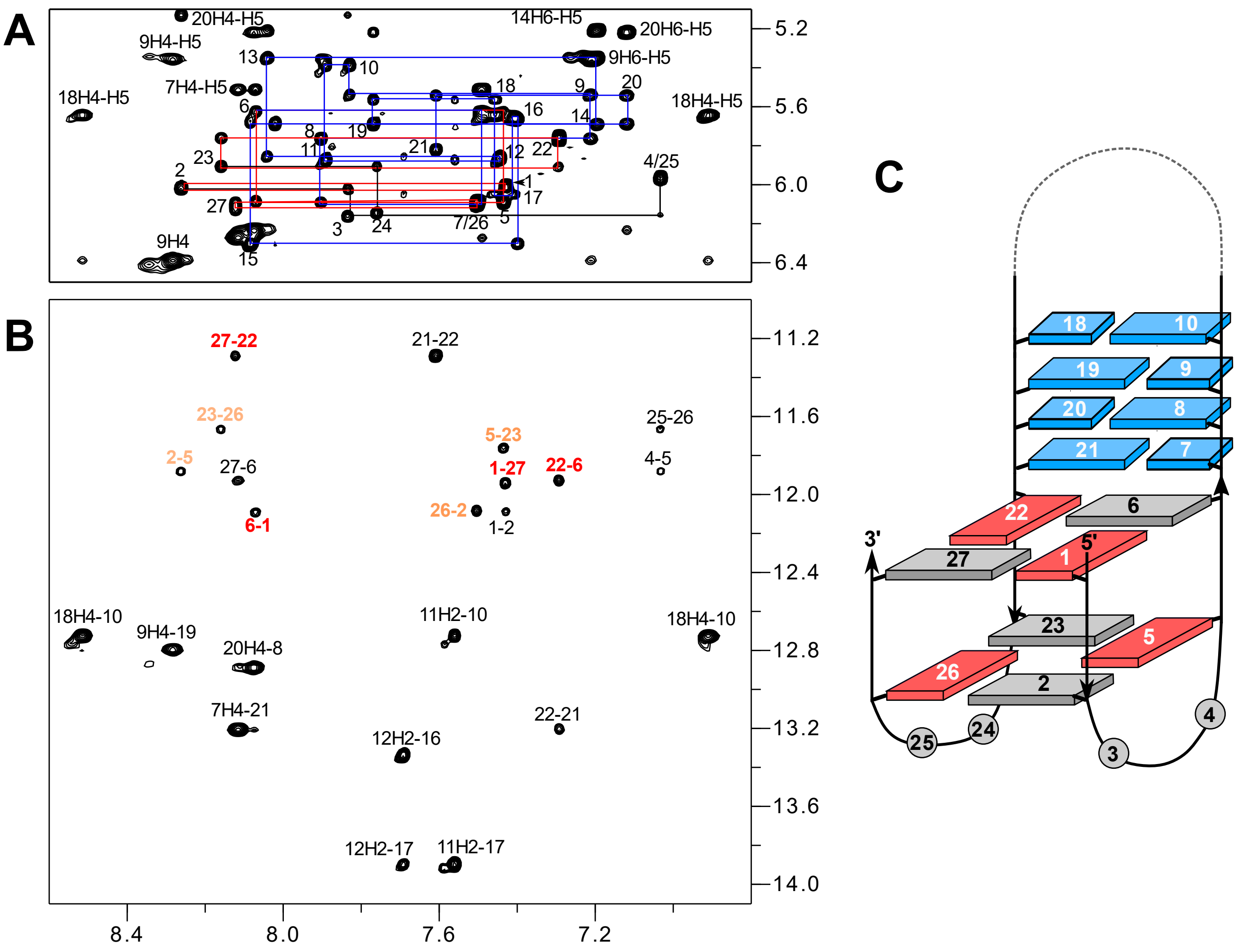

Figure S13. 2D NOESY spectral regions (300 ms mixing time, 20 °C) of *QD2-l* (0.5 mM) showing (A) H8/H6(ω_2)-H1'(ω_1) and (B) H8/6(ω_2)-imino(ω_1) cross-peaks; rectangular NOE patterns of *syn*-*anti* steps typical for the antiparallel strand alignment are indicated by red lines whereas duplex H8/H6-H1' NOE walks are traced by blue lines; intra-tetrad H8(ω_2)-H1(ω_1) cross-peaks in (B) are labeled with colors depending on G-tetrad layer. (C) Schematic representation of *QD2-l*; *syn*-guanosines, *anti*-guanosines, and duplex residues are colored red, grey, and blue, respectively.

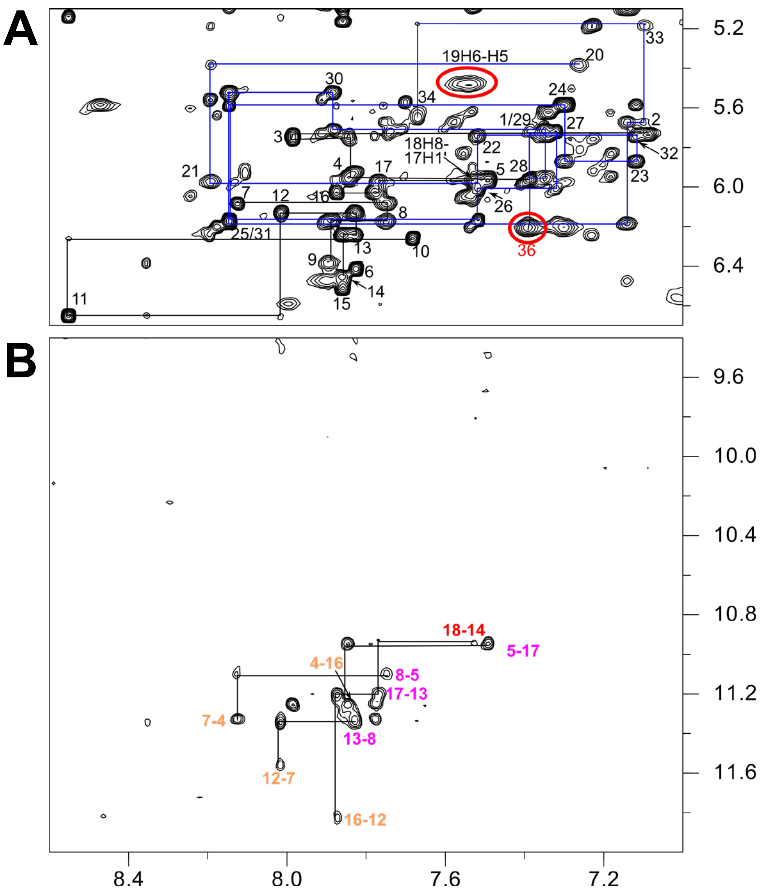

Figure S14. 2D NOESY spectral regions of *QD3-sbl* (0.5 mM) in the presence of 1 eq. cryptolepine. (A) H6/8(ω2)-H1'(ω1) connectivities with continuous networks indicated by blue (duplex domain) and black (quadruplex domain) horizontal and vertical lines; broadened intra-nucleotide H6-H5 and H8-H1' NOE contacts of interfacial C19 and *syn*-G36 are marked by red circles. (B) H6/8(ω2)-H1(ω1) connectivities; intra-tetrad H8(ω2)-H1(ω1) cross-peaks are labeled with colors depending on G-tetrad layer. Spectra were acquired at 30 °C.

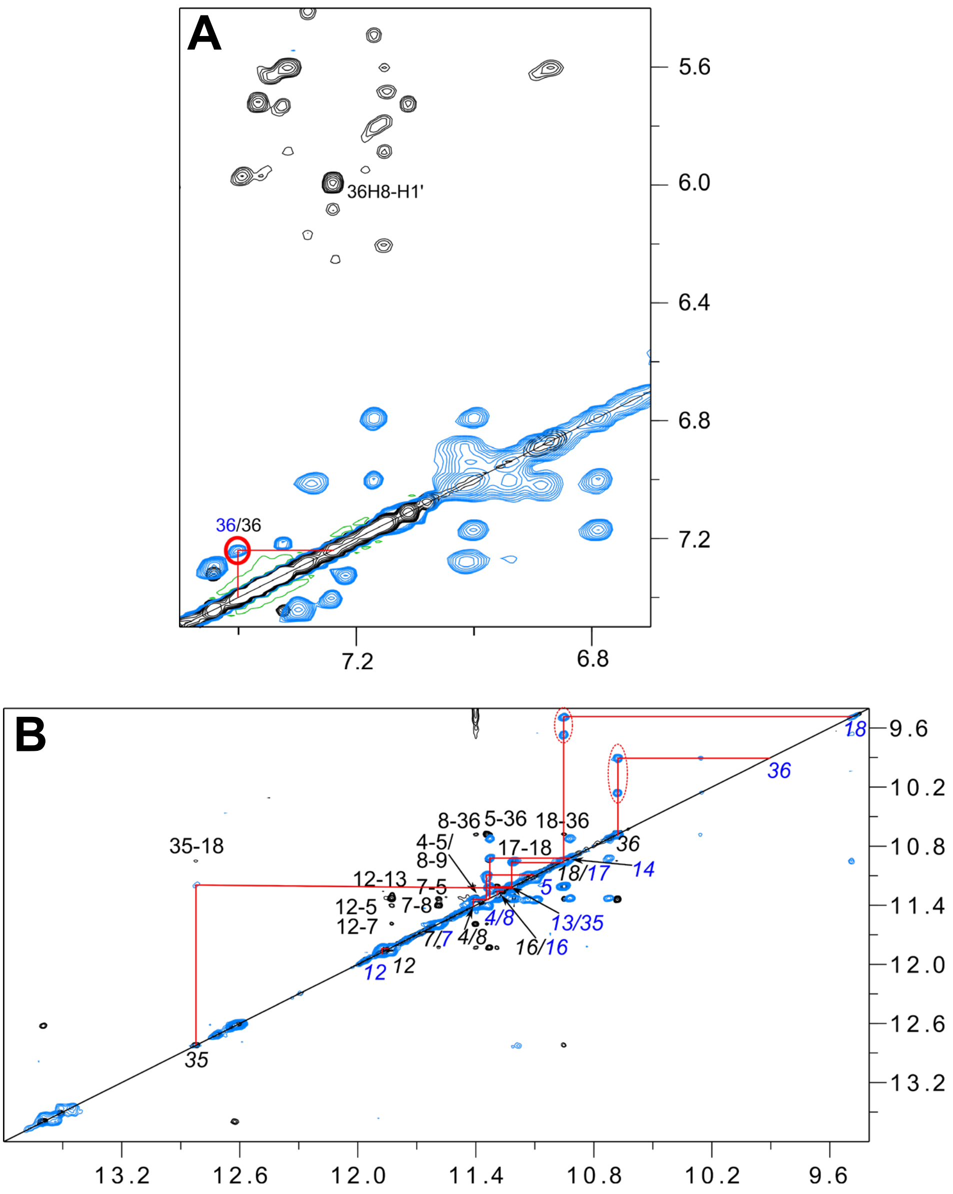

Figure S15. Superimposed NOESY spectral regions of free *QD3-sbl* (300 ms mixing time, black) and corresponding ROESY spectra of *QD3-sbl* in the presence of 0.5 eq. of cryptolepine (blue, only positive contours shown for clarity). (A) H6/8(ω_2)-H1'/H6/H8(ω_1) correlations; G36 H8 of the complex is unambiguously identified through its exchange cross-peak (circled in red) with G36 H8 of the free hybrid. (B) Imino(ω_2)–imino(ω_1) correlations; exchange cross-peaks between free and ligand-bound *QD3-sbl* are traced by red lines with resonances of free and ligand-bound hybrid written in black and blue along the diagonal, respectively; prominent exchange cross-peaks between free and two complexed hybrids are circled in red. Spectra were acquired at 30 °C.

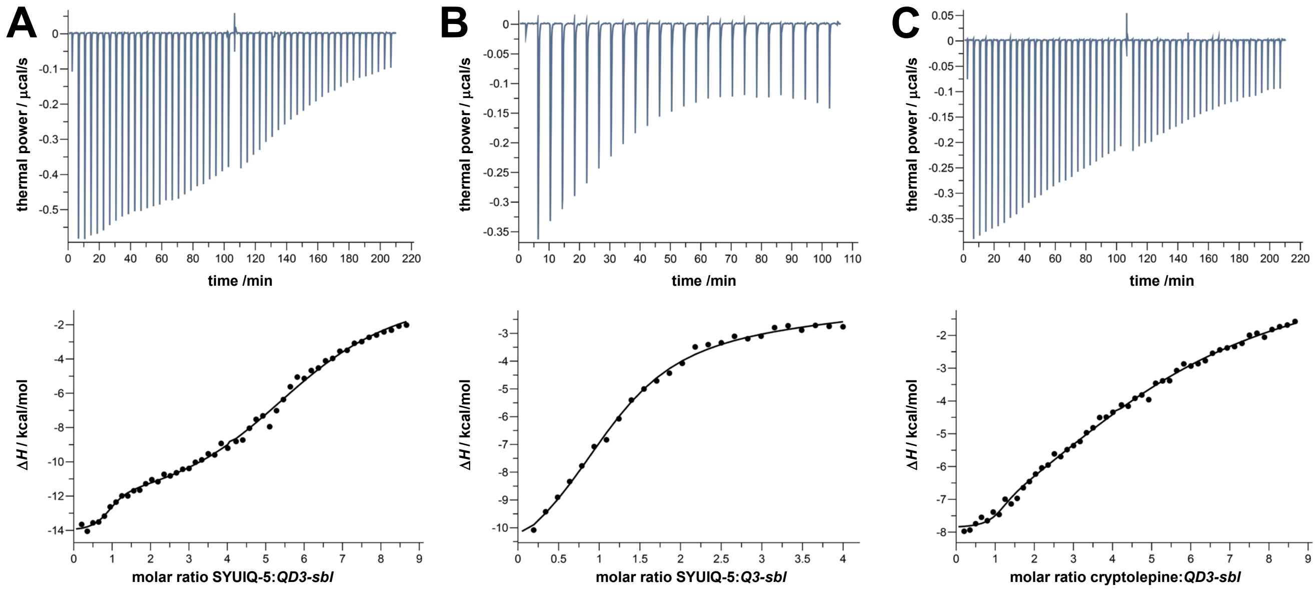

Figure S16. Representative ITC thermograms in 120 mM K^+ buffer at 40 °C for the binding of SYUIQ-5 to *QD3-sbl* (A), SYUIQ-5 to *Q3-sbl* (B), and of cryptolepine to *QD3-sbl* (C). Upper and lower panels show the heat released for each injection step and the blank-corrected normalized heat versus molar ratio, respectively. Curves were fitted with a model of two independent binding sites.

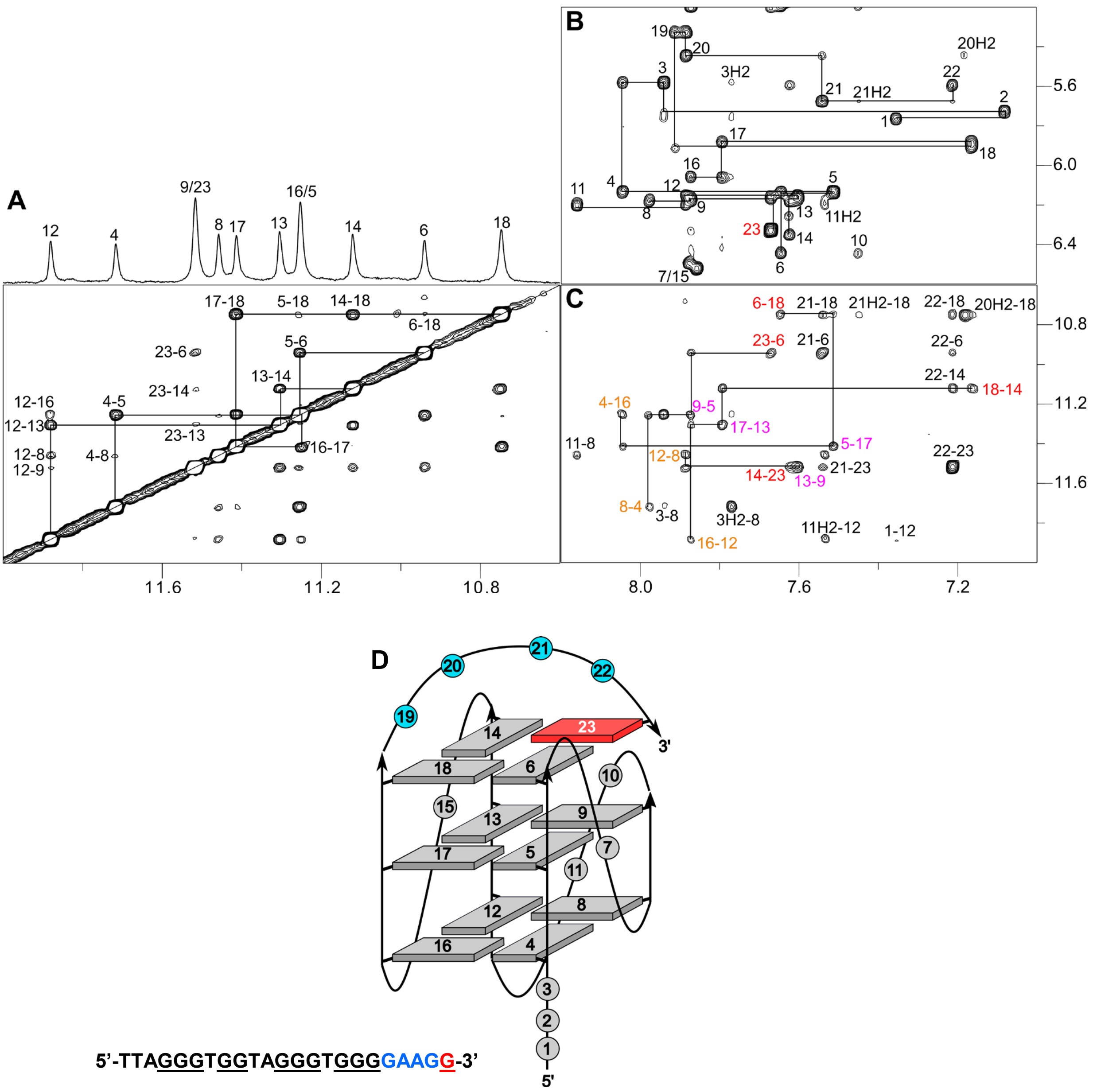

Figure S17. 2D NOESY spectrum (300 ms mixing time, 20 ºC) of *Q3-sbl* (0.2 mM). (A) Imino(ω_2)-imino(ω_1) spectral region with corresponding 1D spectrum; resonances are labeled with residue number. (B) H8/H6(ω_2)-H1'(ω_1) spectral region with an intra-nucleotide H8-H1' contact of *syn*-G23 labeled in red. (C) H8(ω_2)-H1(ω_1) spectral region; intra-tetrad H8(ω_2)-H1(ω_1) cross-peaks are labeled with colors depending on G-tetrad layer. Inter-tetrad connectivities along G-columns are traced by horizontal and vertical lines. (D) Topology and sequence of *Q3-sbl.* The sequence folds into a three-layered parallel quadruplex with a 1:2:1 propeller loop and a 3'-terminal snapback loop arrangement. A vacant site in the second G-column is filled by the 3'-terminal *syn*-G residue.
